# Association of dementia with immunoglobulin G *N*-glycans in a Chinese Han Population

**DOI:** 10.1038/s41514-021-00055-w

**Published:** 2021-02-04

**Authors:** Xiaoyu Zhang, Hui Yuan, Jihui Lyu, Xiaoni Meng, Qiuyue Tian, Yuejin Li, Jie Zhang, Xizhu Xu, Jing Su, Haifeng Hou, Dong Li, Baoliang Sun, Wei Wang, Youxin Wang

**Affiliations:** 1 Department of Epidemiology and Health Statistics, School of Public Health, Beijing Municipal Key Laboratory of Clinical Epidemiology, Capital Medical University, Beijing, 100069 China; 2 Department of Anesthesiology, Sanbo Brain Hospital, Capital Medical University, Beijing, 100095 China; 3 Department of Neurology, The Second Affiliated Hospital of Shandong First Medical University, Tai’an, 271000 China; 4 Center for Cognitive Disorders, Beijing Geriatric Hospital, Beijing, 100095 China; 5 School of public health, Shandong First Medical University & Shandong Academy of Medical Sciences, Tai’an, 271000 China; 6 Department of Geriatrics, Tai’an City Central Hospital, Tai’an, 271000 China; 7 Key Lab of Cerebral Microcirculation in Universities of Shandong, Shandong First Medical University & Shandong Academy of Medical Sciences, Tai’an, 271000 China; 8 School of Medical and Health Sciences, Edith Cowan University, Perth, WA 6027 Australia

**Keywords:** Biomarkers, Inflammation, Risk factors

## Abstract

Immunoglobulin G (IgG) functionality can drastically change from anti- to proinflammatory by alterations in the IgG *N*-glycan patterns. Our previous studies have demonstrated that IgG *N*-glycans associated with the risk factors of dementia, such as aging, dyslipidemia, type 2 diabetes mellitus, hypertension, and ischemic stroke. Therefore, the aim is to investigate whether the effects of IgG *N*-glycan profiles on dementia exists in a Chinese Han population. A case–control study, including 81 patients with dementia, 81 age- and gender-matched controls with normal cognitive functioning (NC) and 108 non-matched controls with mild cognitive impairment (MCI) was performed. Plasma IgG *N*-glycans were separated by ultra-performance liquid chromatography. Fourteen glycan peaks reflecting decreased of sialylation and core fucosylation, and increased bisecting *N*-acetylglucosamine (GlcNAc) *N*-glycan structures were of statistically significant differences between dementia and NC groups after controlling for confounders (*p* < 0.05; *q* < 0.05). Similarly, the differences for these 14 initial glycans were statistically significant between AD and NC groups after adjusting for the effects of confounders (*p* < 0.05; *q* < 0.05). The area under the receiver operating curve (AUC) value of the model consisting of GP8, GP9, and GP14 was determined to distinguish dementia from NC group as 0.876 [95% confidence interval (CI): 0.815–0.923] and distinguish AD from NC group as 0.887 (95% CI: 0.819–0.936). Patients with dementia were of an elevated proinflammatory activity via the significant changes of IgG glycome. Therefore, IgG *N*-glycans might contribute to be potential novel biomarkers for the neurodegenerative process risk assessment of dementia.

## Introduction

Dementia is a major global challenge for health and social care in the 21st century. It occurs mainly in subjects older than 65 years, making them gradually lose their abilities and become more dependent^1^. Alzheimer disease (AD) is the most common form of dementia, accounting for approximately 60–80% of all types^1^. The concept of mild cognitive impairment (MCI) is as an intermediate state between normal cognition and dementia; nevertheless, the boundary between the MCI and dementia is gray^2^. Research has found that potentially modifiable risk factors of worldwide dementia, mainly vascular risk factors, account for 40% (hypertension, obesity, diabetes, later-life smoking, depression, physical inactivity, etc.)^3,4^. Besides, increasing age is also one of the strongest known risk factors for dementia^5,6^. During the process of senescence, cells secrete a large number of inflammatory factors and chemokines, such as interleukin-6 (IL-6), interleukin-1 beta (IL-1β), and tumor necrosis factor-alpha (TNF-α), which can produce chronic inflammatory responses^5,7^. Accumulating evidence has suggested that inflammatory responses make a valuable contribution towards the neurodegenerative cascades of dementia and MCI, and several markers were found to be able to accurately detect the disease severity and progression^8–12^. The mechanisms of dementia, however, are still largely unknown.

Glycosylation is a complex post-translational modification during gene expression involved in more than half of all mammalian proteins^13^. Glycan binding in functional proteins has a crucial role in the biological processes of molecular recognition and adhesion, cell signaling or immunological response^14,15^. Hence, molecular defects in glycan synthesis are recognized as direct causes of an increasing number of diseases^16,17^. Immunoglobulin G (IgG) exerts the role in the immune system the body^18^. In terms of its characteristic of structure with conserved *N*-glycosylation in Fc, so IgG is one of the mostly suitable choices for glycoproteins research^19^. The attachment of different glycans displays conformational flexibility and plasticity which can be dramatically influence on IgG effector functions^15^. In particular, the glycan portion of IgG critically affects immune effector functions mediated by the Fc receptor and complement C1q^20^. It modulates in relation to current physiological conditions, but can also change in an “on and off mode” during acute inflammation^21^. Hence, changes in IgG glycosylation not only participate in many kinds of inflammatory diseases^22–25^, but also the molecular mechanism may give rise to the promotion of inflammation^21,26,27^.

Considering the fact that the inflammatory role of IgG *N*-glycosylation and its association with the risk factors of dementia [such as aging, central obesity, dyslipidemia, type 2 diabetes mellitus (T2DM), hypertension, and ischemic stroke]^28–34^, we hypothesized that these associations might provide a possible explanation for the pathogenesis of dementia in a Chinese Han population. In addition, previous study provided the evidence in European ancestry individuals that a lower abundance of complex galactosylation and sialylation played a role in the development of AD^35^. Therefore, the aim is to explore whether the changes in IgG glycosylation can identify specific alterations for dementia progress in a Chinese Han population and reveal dementia potential pathogenesis. Meanwhile, we investigate the associations between IgG *N*-glycan profiles and inflammation factors including anti-inflammatory (IL-4 and IL-10) and proinflammatory factors [IL-1β, IL-6, CRP, TNF-α, and interferon-gamma (IFN-γ)], which may further explain the role of IgG glycosylation in dementia.

## Results

### Participants’ characteristics

**Fig. 1:**
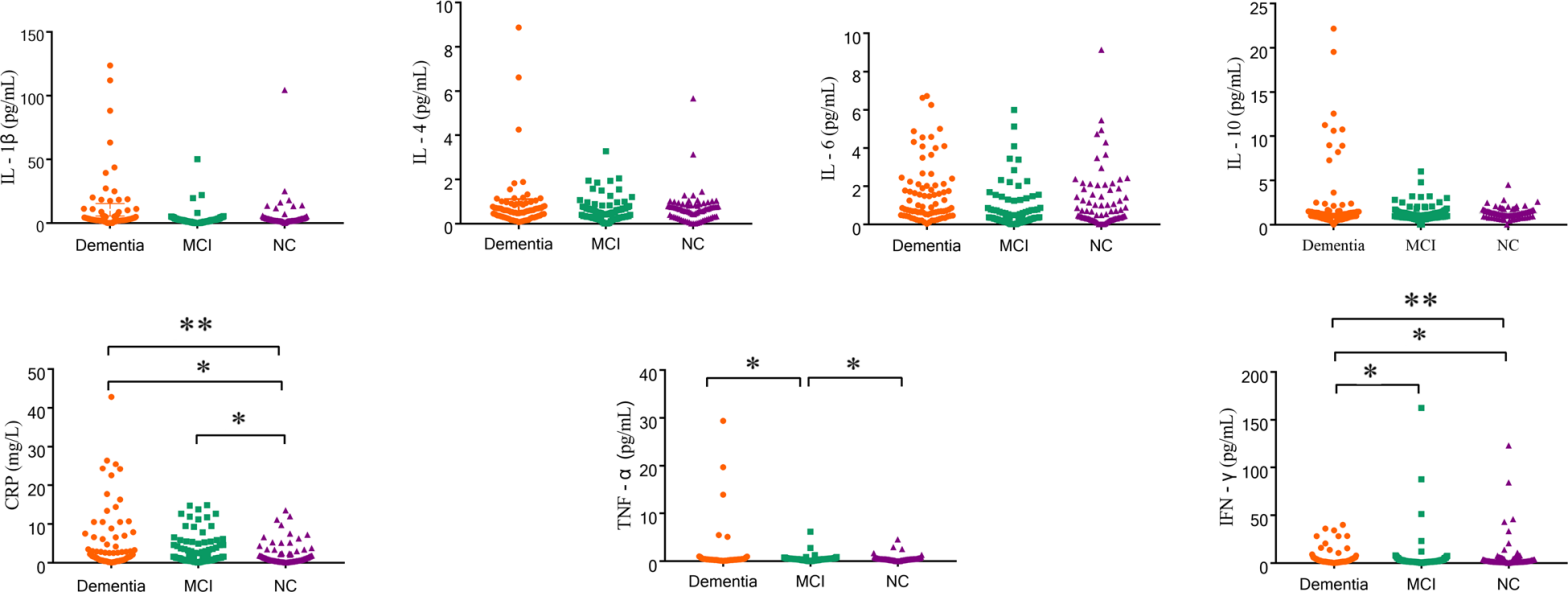
The scatter plots of different levels of inflammatory factors among dementia, MCI, and NC groups. The scatter plots show the different levels of anti-inflammatory and proinflammatory factors in dementia (orange), MCI (green), and NC (purple) groups. Anti-inflammatory factors include IL-4 and IL-10, while proinflammatory factors include IL-1β, IL-6, CRP, IFN-γ, and TNF-α. *There were significant differences between the two groups (*p* < 0.017); **There are significant differences for the trend tests (*p* < 0.05). NC normal cognitive functioning, MCI mild cognitive impairment, IL-4 interleukin-4, IL-10 interleukin-10, IL-1β interleukin-1 beta, CRP C-reactive protein, IFN-γ Interferon-gamma, TNF-α tumor necrosis factor alpha.

In total, 81 patients with dementia, 81 age- and gender-matched participants with NC and 108 non-matched participants with MCI were included in the present study. The 81 dementia participants consisted of 47 (58.00%) AD, 18 (22.20%) vascular dementia (VaD), and 16 (19.80%) other type of dementia. The differences of demographic characteristics and inflammatory factors among three groups were presented in Table 1 and Fig. 1. The median ages for three groups were 82, 80, and 76 years, respectively. The number of females was generally higher than males for each group, but it was not statistically significant among three groups. The values of TC and LDL-C in the dementia group were significantly lower than these in the NC group (*p* < 0.017). In addition, age in the dementia group was significantly higher than that in the MCI group, whereas values of systolic blood pressure (SBP) and diastolic blood pressure (DBP) were lower than these in the MCI group (*p* < 0.017). The levels of SBP, DBP, TC, and LDL-C of MCI group were significantly higher than NC group (*p* < 0.017).

**Table 1:** Characteristics of the study subjects.

Parameters	NC (*n* = 81)	MCI (*n* = 108)	Dementia (*n* = 81)							
			Total	*p*	AD (*n* = 47)	*p*	VaD (*n* = 18)	*p*	OD (*n* = 16)	*p*
Gender (male/female)	38/43	42/66	38/43	0.617	22/25	0.392	8/10	0.537	8/8	0.542
Age, years	80.00 (73.50–85.00)	76.00 (73.00–81.00)	82.00 (75.50–85.00)^#&^	<0.001	82.00 (77.00–82.00)	<0.001	79.50 (74.25–84.25)	0.039	81.00 (74.00–84.75)	0.039
BMI, kg/m^2^	23.24 (20.96–25.62)	23.81 (21.52–26.70)	22.50 (20.84–24.45)	0.072	22.46 (20.76–23.81)	0.053	24.10 (20.89–26.96)	0.580	22.65 (21.69–24.01)	0.372
Levels of education, *n* (%)				<0.001		<0.001		0.001		<0.001
Illiteracy	10 (12.80)	45 (42.90)	8 (10.70)^#$^		7 (15.20)		0 (0.00)		1 (6.30)	
Primary school	30 (38.50)	26 (24.80)	20 (27.70)		9 (19.60)		9 (69.20)		2 (12.50)	
Middle school	16 (20.50)	12 (11.40)	9 (12.00)		3 (6.50)		2 (15.40)		4 (25.00)	
High school	6 (7.70)	13 (12.40)	5 (6.70)		4 (8.70)		0 (0.00)		1 (6.30)	
College or University or above	16 (20.50)	9 (8.60)	33 (44.00)		23 (50.00)		2 (15.40)		8 (50.00)	
Smoking, *n* (%)	23 (28.40)	28 (25.90)	23 (29.90)	0.832	10 (21.70)	0.851	7 (46.70)	0.250	6 (37.50)	0.871
Drinking, *n* (%)	21 (25.90)	28 (25.90)	16 (19.80)	0.554	7 (14.90)	0.284	5 (27.80)	0.986	4 (25.00)	0.997
Habit of salt intake				<0.001		0.180		0.143		0.199
High	23 (28.40)	37 (34.30)	8 (9.90)^&$^		4 (8.50)		3 (16.70)		1 (6.30)	
Normal	20 (24.70)	36 (33.30)	58 (71.60)		37 (78.70)		7 (38.90)		14 (87.50)	
Low	38 (46.90)	35 (32.40)	15 (18.50)		6 (12.80)		8 (44.40)		1 (6.30)	
Family history of dementia, *n* (%)	2 (2.50)	6 (5.60)	7 (8.60)	0.260	5 (10.60)	0.150	1 (5.60)	0.570	1 (6.30)	0.552
Hypertension, *n* (%)	51 (63.00)	67 (62.00)	58 (71.60)	0.347	32 (68.10)	0.606	15 (83.30)	0.209	11 (68.80)	0.992
Diabetes, *n* (%)	18 (22.20)	36 (33.30)	28 (34.60)	0.160	18 (38.30)	0.113	7 (38.90)	0.167	3 (18.80)	0.170
Depression, *n* (%)	2 (2.50)	2 (1.90)	7 (8.60)	0.084	4 (8.50)	0.094	1 (5.60)	0.639	2 (12.50)	0.060
Ischemic stroke, *n* (%)	20 (24.70)	30 (27.80)	47 (58.00)^&$^	<0.001	26 (55.30)	0.002	13 (72.20)	<0.001	8 (50.00)	0.037
Malignant tumor, *n* (%)	2 (2.50)	1 (0.90)	5 (6.20)	0.132	3 (6.40)	0.141	1 (5.60)	0.379	1 (6.30)	0.326
Cardiovascular disease, *n* (%)	22 (27.20)	37 (34.30)	27 (33.30)	0.551	18 (38.30)	0.385	4 (22.20)	0.423	5 (31.30)	0.582
FBG, mmol/L	5.09 (4.65–6.19)	5.48 (4.73–6.66)	5.10 (4.50–5.90)	0.049	5.20 (4.50–5.90)	0.165	4.15 (2.19–5.00)	0.003	5.55 (4.80–5.88)	0.219
SBP, mmHg	137.00 (125.00–150.00)	148.50 (131.00–164.25)	132.00 (124.00–144.50)^#&^	<0.001	134.00 (122.00–144.00)	<0.001	138.50 (123.00–161.00)	0.007	132 (124.25–139.25)	<0.001
DBP, mmHg	75.00 (69.00–82.00)	79.00 (72.75–86.25)	72.00 (67.50–80.00)^#&^	<0.001	70.00 (66.00–78.00)	<0.001	79.00 (71.75–84.50)	0.048	70.50 (66.25–78.00)	0.004
TC, mmol/L	4.36 (3.80–5.01)	4.96 (4.01–5.97)	4.37 (3.59–5.58)^#^	0.011	4.08 (3.56–5.58)	0.005	5.32 (4.42–6.29)	0.002	4.25 (3.43–5.37)	0.007
TG, mmol/L	1.24 (0.89–2.10)	1.25 (0.90–1.72)	1.30 (0.98–2.32)	0.470	1.20 (0.89–1.77)	0.922	2.85 (1.31–3.96)	0.002	1.21 (0.96–1.61)	0.961
HDL-C, mmol/L	1.20 (0.96–1.45)	1.22 (1.05–1.44)	1.12 (0.93–1.31)	0.086	1.12 (0.91–1.29)	0.076	1.16 (0.79–1.96)	0.758	1.12 (0.99–1.23)	0.399
LDL-C, mmol/L	2.23 (1.72–2.61)	2.65 (2.05–3.30)	2.35 (1.78–3.19)^#^	0.005	2.35 (1.80–3.31)	0.004	1.71 (0.89–3.05)	0.001	2.61 (2.09–3.39)	0.001

Data were expressed median (*P*_25_ – *P*_75_), or *n* (%). *NC* normal cognitive functioning, *MCI* mild cognitive impairment, *BMI* body mass index, *FBG* fasting blood glucose, *SBP* systolic blood pressure, *DBP* diastolic blood pressure, *TC* total cholesterol, *TG* total triglycerides, *HDL-C* high-density lipoprotein cholesterol, *LDL-C* low-density lipoprotein cholesterol, *AD* Alzheimer’s disease, *VaD* vascular dementia, *OD* other type of dementia.

^#^*p* < 0.017, MCI group compared with NC group.

^$^*p* < 0.017, Dementia group compared with MCI group.

^&^*p* < 0.017, Dementia group compared with NC group.

The levels of CRP, TNF-α, and IFN-γ were of significant differences among three groups, whereas the levels of IL-1β, IL-4, IL-6, and IL-10 were not of significant differences (*p* < 0.05) (Fig. 1).

### IgG glycome compositions

**Fig. 2:**
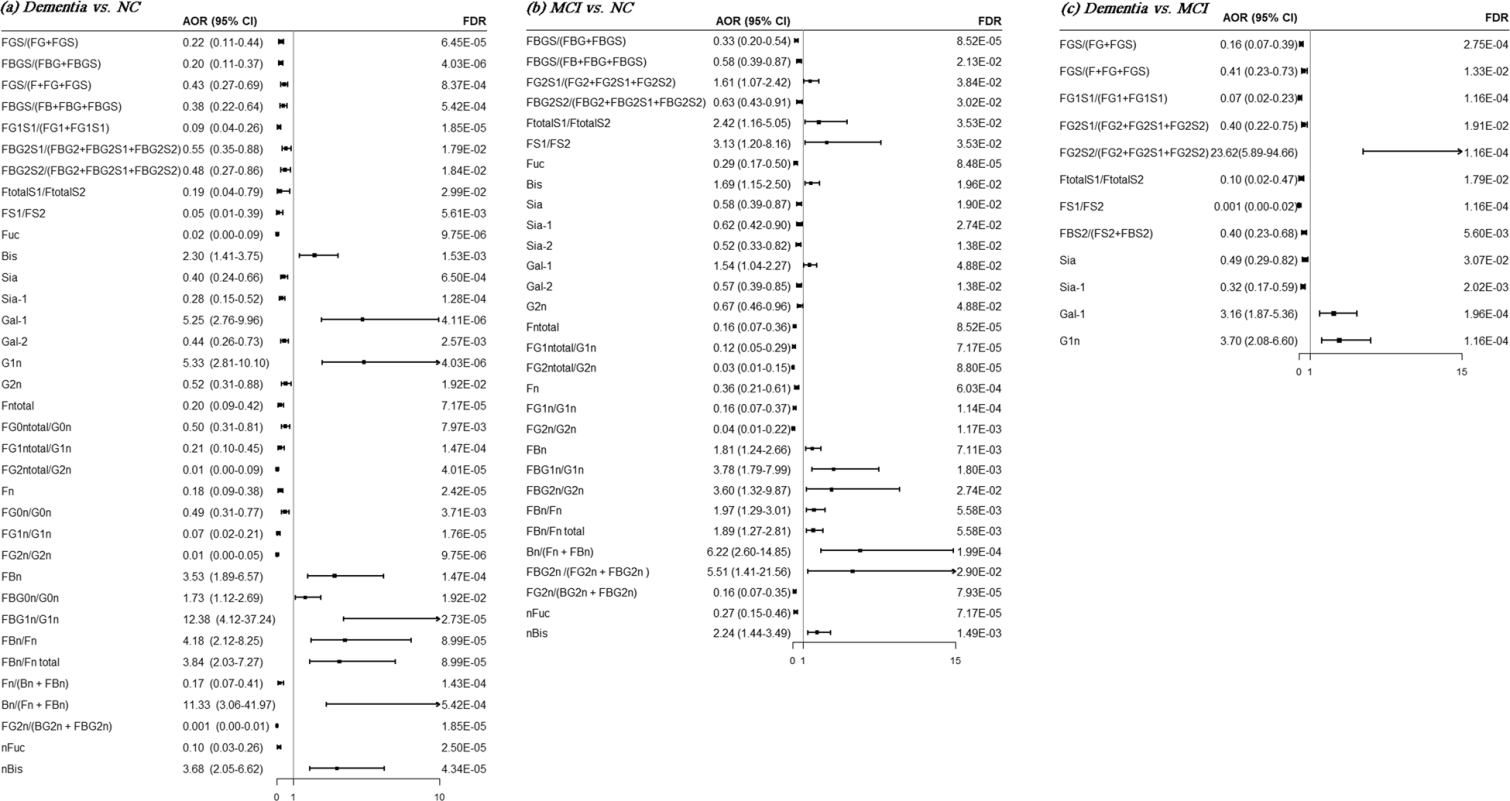
Forest plots of the associations of the derived traits with MCI and dementia. Forest plots (**a**–**c**) show the odds ratios (ORs, black solid square) with a horizontal lines representing 95% confidence intervals (CIs) for derived traits with MCI and dementia. Each of statistically significant derived traits included study is represented by one row in the plots. The multiple logistic regression analysis was performed after adjusting for age, sex, BMI, levels of education, history of malignant tumor, habit of salt intake, ischemic stroke, diabetes, hypertension, and dyslipidemia (adjusting for the above effects other than age and sex for dementia vs. NC). **a** Dementia vs. NC, **b** MCI vs. NC, and **c** Dementia vs. MCI. NC normal cognitive functioning, AD Alzheimer’s disease.

Of the 22 initial glycans, 18 were significantly skewed (*p* < 0.05) (Supplementary Table 3). The comparisons of initial glycans compositions among dementia, MCI and NC groups were shown in Table 2, while these of derived traits were shown in Supplementary Table 4. After adjusting for BMI, levels of education, history of malignant tumor, habit of salt intake, ischemic stroke, diabetes, hypertension, and dyslipidemia in the logistic regression, 14 initial glycans (increased relative abundance of GP1, GP2, GP5, GP7, GP8, GP9, GP10, GP12, GP13, GP15, and GP22 as well as reduced relative abundance of GP14, GP16, and GP18) remained significantly different between dementia and NC groups (*p* < 0.05; *q* < 0.05) (Table 3 and Supplementary Fig. 1). Derived glycan traits were of significant differences in patients with dementia compared to NC group, primarily reflecting the decreased of sialylation and core fucosylation, as well as the increased bisecting *N*-acetylglucosamine (GlcNAc) *N*-glycan structures in dementia patients (Fig. 2 and Supplementary Table 5).

**Table 2:** The levles of initial glycans from NC, MCI, and dementia patients.

GPs	NC (*n* = 81)	MCI (*n* = 108)	Dementia (*n* = 81)	p*
	Median (*P*_25_–*P*_75_)	Median (*P*_25_–*P*_75_)	Median (*P*_25_–*P*_75_)	
GP1	0.05 (0.02–0.20)	0.37 (0.16–0.60)	0.33 (0.22–0.57)^#$^	<0.001
GP2	0.28 (0.10–0.45)	0.37 (0.17–0.73)	0.45 (0.24–0.78)^#$^	0.001
GP4	42.02 (34.75–47.92)	41.59 (34.85–47.32)	39.16 (34.28–45.70)^a^	0.575
GP5	0.00 (0.00–0.02)	0.05 (0.01–0.17)	0.20 (0.07–0.36)^#&$^	<0.001
GP6	6.22 (4.76–7.78)	6.23 (5.17–7.52)	6.51 (5.21–7.73)^a^	0.937
GP7	0.01 (0.00–0.05)	0.14 (0.05–0.27)	0.15 (0.05–0.40)^#$^	<0.001
GP8	11.53 (10.35–12.87)	12.35 (10.02–13.91)	14.46 (13.11–15.57)^#&^	<0.001
GP9	2.97 (1.72–4.64)	3.35 (2.00–5.06)	4.29 (3.29–5.58)^#&^	<0.001
GP10	0.73 (0.52–1.13)	1.11 (0.57–1.93)	2.12 (1.19–2.87)^#&$^	<0.001
GP11	0.08 (0.06–0.23)	0.31 (0.14–0.60)	0.10 (0.05–0.31)^&$^	<0.001
GP12	0.14 (0.04–0.32)	0.16 (0.08–0.31)	0.32 (0.17–0.58)^#&^	<0.001
GP13	0.14 (0.04–0.32)	0.31 (0.15–0.76)	0.36 (0.25–0.73)^#$^	<0.001
GP14	13.73 (11.43–17.54)	13.02 (8.74–16.56)	11.24 (7.89–14.26)^a#^	0.001
GP15	0.11 (0.06–0.18)	0.21 (0.11–0.43)	0.45 (0.23–0.68)^#&$^	<0.001
GP16	3.97 (2.96–4.64)	3.66 (2.99–4.49)	2.93 (2.34–3.46)^a^^#&^	<0.001
GP17	0.83 (0.51–1.20)	0.56 (0.34–0.90)	0.75 (0.57–1.12)^&$^	<0.001
GP18	10.52 (7.73–13.29)	9.27 (7.75–12.12)	7.2 (5.89–9.06)^#&^	<0.001
GP19	1.76 (1.28–2.24)	1.86 (1.31–2.42)	1.77 (1.29–2.12)	0.429
GP21	0.23 (0.17–0.31)	0.18 (0.13–0.26)	0.32 (0.23–0.53)^#&$^	<0.001
GP22	0.07 (0.46–0.14)	0.14 (0.07–0.27)	0.11 (0.08–0.24)^#$^	<0.001
GP23	0.67 (0.42–1.03)	0.53 (0.34–0.82)	0.84 (0.50–1.10)^&$^	<0.001
GP24	0.82 (0.59–1.33)	0.83 (0.49–1.21)	0.76 (0.47–1.05)	0.449

*NC* normal cognitive functioning, *MCI* mild cognitive impairment.

*p < 0.05 was considered statistically significant.

^#^*p* < 0.017, Dementia group compared with NC group.

^$^*p* < 0.017, MCI group compared with NC group.

^&^*p* < 0.017, Dementia group compared with MCI group.

^a^Analysis of variance (ANOVA).

**Table 3:** Associations of the normalized initial glycans.

GPs	Dementia vs. NC			Dementia vs. MCI			MCI vs. NC		
	OR (95% CI)^#^	*p*-adjusted	q*	OR (95% CI)^#^	*p*-adjusted	q*	OR (95% CI)^#^	*p*-adjusted	q*
GP1	6.78 (3.01–15.29)	3.91E−06	1.72E−05	0.80 (0.54–1.18)	2.59E−01	3.97E−01	9.53 (4.24–21.41)	4.73E−08	1.04E−06
GP2	1.64 (1.07–2.52)	2.42E−02	3.80E−02	0.91 (0.62–1.34)	6.19E−01	6.81E−01	1.96 (1.25–3.08)	3.36E−03	8.21E−03
GP4	0.84 (0.58–1.22)	3.63E−01	4.70E−01	0.81 (0.55–1.21)	3.01E−01	4.13E−01	1.12 (0.78–1.60)	5.48E−01	5.74E−01
GP5	32.78 (8.31–129.27)	6.19E−07	4.50E−06	1.34 (0.85–2.11)	2.03E−01	3.67E−01	8.16 (2.95–22.58)	5.29E−05	2.91E−04
GP6	1.16 (0.78–1.71)	4.65E−01	5.11E−01	0.73 (0.49–1.09)	1.27E−01	2.53E−01	1.29 (0.94–1.77)	1.12E−01	1.36E−01
GP7	4.71 (2.29–9.67)	2.46E−05	6.77E−05	1.17 (0.79–1.73)	4.25E−01	5.43E−01	6.58 (3.02–14.34)	2.14E−06	2.35E−05
GP8	6.02 (3.05–11.89)	2.31E−07	3.10E−06	3.46 (2.02–5.94)	6.58E−06	1.45E−04	1.03 (0.74–1.43)	8.79E−01	8.79E−01
GP9	2.06 (1.37–3.10)	4.94E−04	1.09E−03	1.65 (1.12–2.44)	1.14E−02	3.57E−02	1.37 (0.98–1.93)	6.87E−02	8.89E−02
GP10	4.87 (2.66–8.90)	2.82E−07	3.10E−06	1.45 (1.00–2.10)	4.94E−02	1.21E−01	2.51 (1.54–4.11)	2.40E−04	1.06E−03
GP11	1.34 (0.62–2.90)	4.56E−01	5.11E−01	0.44 (0.25–0.77)	3.77E−03	1.38E−02	4.37 (1.95–9.79)	3.34E−04	1.22E−03
GP12	2.24 (1.39–3.63)	9.86E−04	1.97E−03	1.78 (1.09–2.88)	2.02E−02	5.55E−02	1.41 (0.89–2.24)	1.48E−01	1.71E−01
GP13	6.65 (2.55–17.36)	1.09E−04	2.66E−04	1.16 (0.80–1.68)	4.45E−01	5.43E−01	4.89 (2.32–10.30)	3.02E−05	2.21E−04
GP14	0.49 (0.30–0.78)	2.78E−03	5.10E−03	1.02 (0.69–1.50)	9.26E−01	9.26E−01	0.62 (0.43–0.88)	7.08E−03	1.56E−02
GP15	8.10 (3.53–18.61)	8.18E−07	4.50E−06	1.11 (0.78–1.57)	5.80E−01	6.72E−01	3.28 (1.65–6.53)	7.09E−04	2.23E−03
GP16	0.34 (0.21–0.55)	1.09E−05	4.00E−05	0.38 (0.23–0.63)	1.74E−04	1.28E−03	0.70 (0.50–0.99)	4.11E−02	6.45E−02
GP17	0.96 (0.63–1.45)	8.30E−01	8.33E−01	1.32 (0.85–2.05)	2.17E−01	3.67E−01	0.63 (0.42–0.93)	2.12E−02	3.58E−02
GP18	0.36 (0.22–0.57)	2.38E−05	6.77E−05	0.38 (0.22–0.66)	5.89E−04	3.24E−03	0.65 (0.46–0.92)	1.38E−02	2.52E−02
GP19	0.68 (0.39–1.20)	1.83E−01	2.52E−01	0.58 (0.30–1.15)	1.19E−01	2.53E−01	0.85 (0.62–1.17)	3.21E−01	3.53E−01
GP21	1.60 (0.98–2.62)	6.18E−02	9.06E−02	4.49 (2.15–9.38)	6.49E−05	7.14E−04	0.58 (0.33–1.00)	4.99E−02	7.32E−02
GP22	3.59 (1.40–9.19)	7.76E−03	1.31E−02	1.04 (0.69–1.56)	8.53E−01	8.93E−01	4.60 (1.85–11.43)	1.00E−03	2.75E−03
GP23	0.87 (0.64–1.20)	4.07E−01	4.98E−01	2.19 (1.31–3.64)	2.62E−03	1.15E−02	0.57 (0.37–0.87)	9.40E−03	1.88E−02
GP24	1.04 (0.73–1.48)	8.33E−01	8.33E−01	1.21 (0.86–1.70)	2.71E−01	3.97E−01	0.69 (0.47–1.02)	6.37E−02	8.75E−02

*q < 0.05: significant after correction for FDR (false discovery rate).

*NC* normal cognitive functioning, *MCI* mild cognitive impairment.

^#^Adjusting for the effects of age, sex, BMI, levels of education, history of malignant tumor, habit of salt intake, ischemic stroke, diabetes, hypertension and dyslipidemia (adjusting for the above effects other than age and sex for dementia vs. NC).

*p* < 0.05 was considered statistically significant.

Similarly, these 14 initial glycans were significantly different between AD and NC groups after adjusting for the effects of above confounders (*p* < 0.05; *q* < 0.05) (Supplementary Table 6 and Supplementary Fig. 1). In addition, 7 initial glycans (increased relative abundance of GP8, GP9, GP12, GP21, and GP23 as well as reduced relative abundance of GP16 and GP18) were of significant differences between dementia and MCI groups after adjusting these confounders (*p* < 0.05; *q* < 0.05) (Table 3). Twelve IgG glycans (increased relative abundance of GP1, GP2, GP5, GP7, GP10, GP11, GP13, GP15, and GP22 as well as reduced relative abundance of GP14, GP17, and GP23) were of significant differences between MCI and NC groups after adjusting for these confounders (*p* < 0.05; *q* < 0.05) (Table 3).

### The dimension reduction and diagnostic values of IgG *N*-glycans

**Fig. 3:**
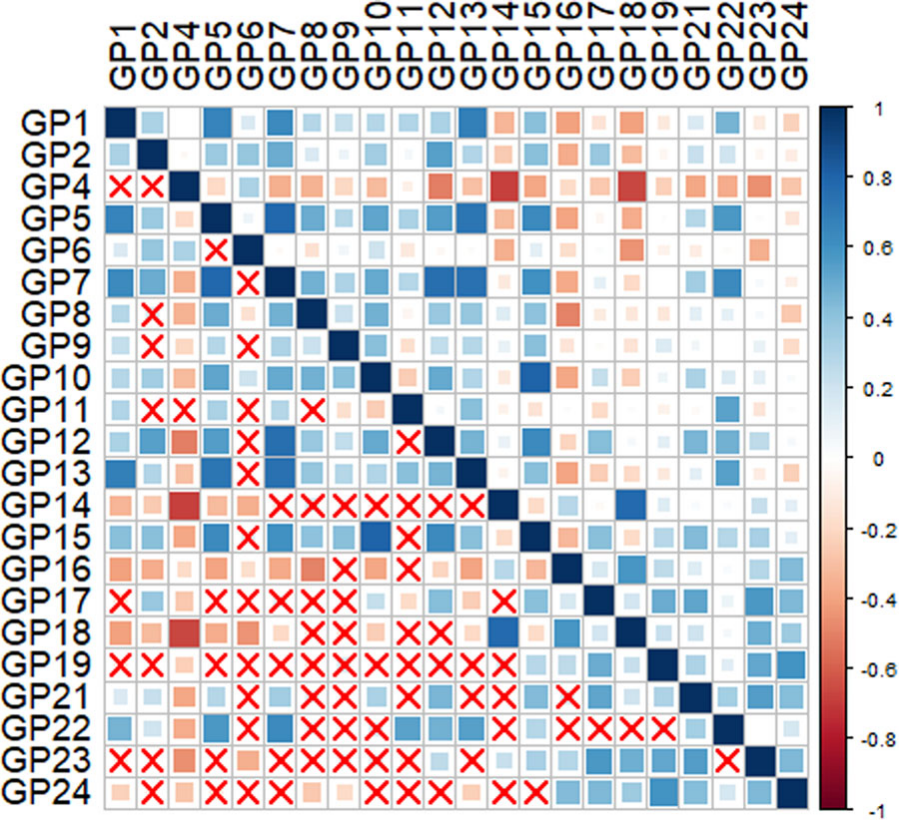
The correlation coefficients in independent glycans. Statistically significant associations between two glycans were shown, *p* < 0.05, while the insignificant correlation coefficients were shown using symbol (“×”) in the boxes. The positive correlations are represented by blue color, while negative correlations are represented by red color.

**Fig. 4:**
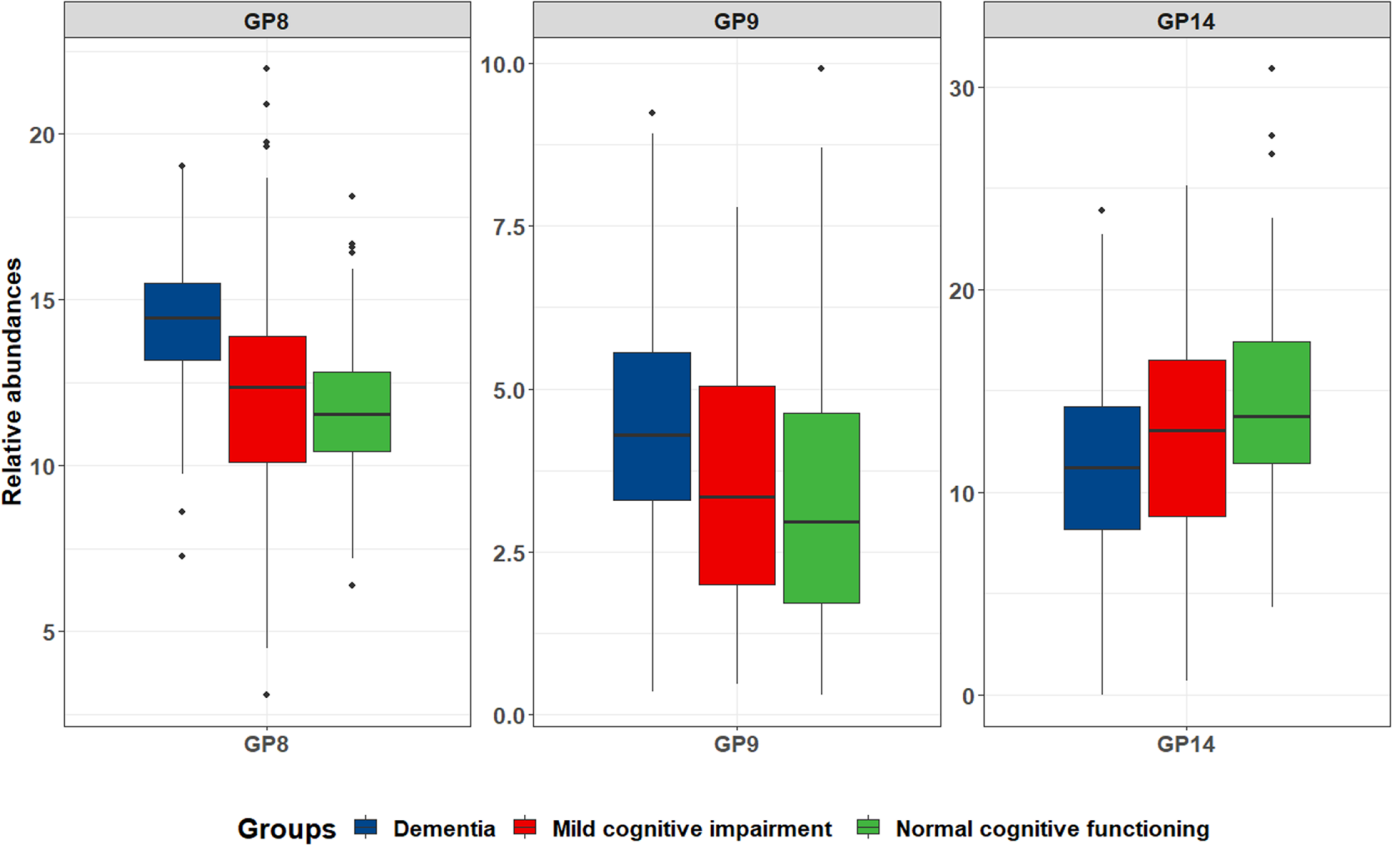
The distribution of a panel of biomarkers (GP8, GP9 and GP14) within the dementia, mild cognitive impairment (MCI), and normal cognitive functioning (NC) groups. The box plots show the distribution of GP8, GP9, and GP14 in dementia (blue), MCI (red), and NC (green) groups. The following values correspond to center line, upper/lower bounds, and whisker max/min for dementia, MCI, and NC groups, respectively: **GP8**: center line (median): 14.46, 12.35, 11.53; lower/upper bounds (*P*_25_/*P*_75_): 13.11/15.57, 10.02/13.91, 10.35/12.87; whiskers max/min: 19.01/7.27, 21.96/3.09, 18.10/6.38; **GP9**: center line (median): 4.29, 3.35, 2.97; lower/upper bounds (*P*_25_/*P*_75_): 3.29/5.58, 2.00/5.06, 1.72/4.64; whiskers max/min: 9.23/0.36, 7.78/0.47, 9.91/0.31; **GP14**: center line (median): 11.24, 13.02, 13.73; lower/upper bounds (*P*_25_/*P*_75_): 7.89/14.26, 8.74/16.56, 11.43/17.54; whiskers max/min: 23.87/0.01, 25.14/0.69, 30.89/4.34.

**Fig. 5:**
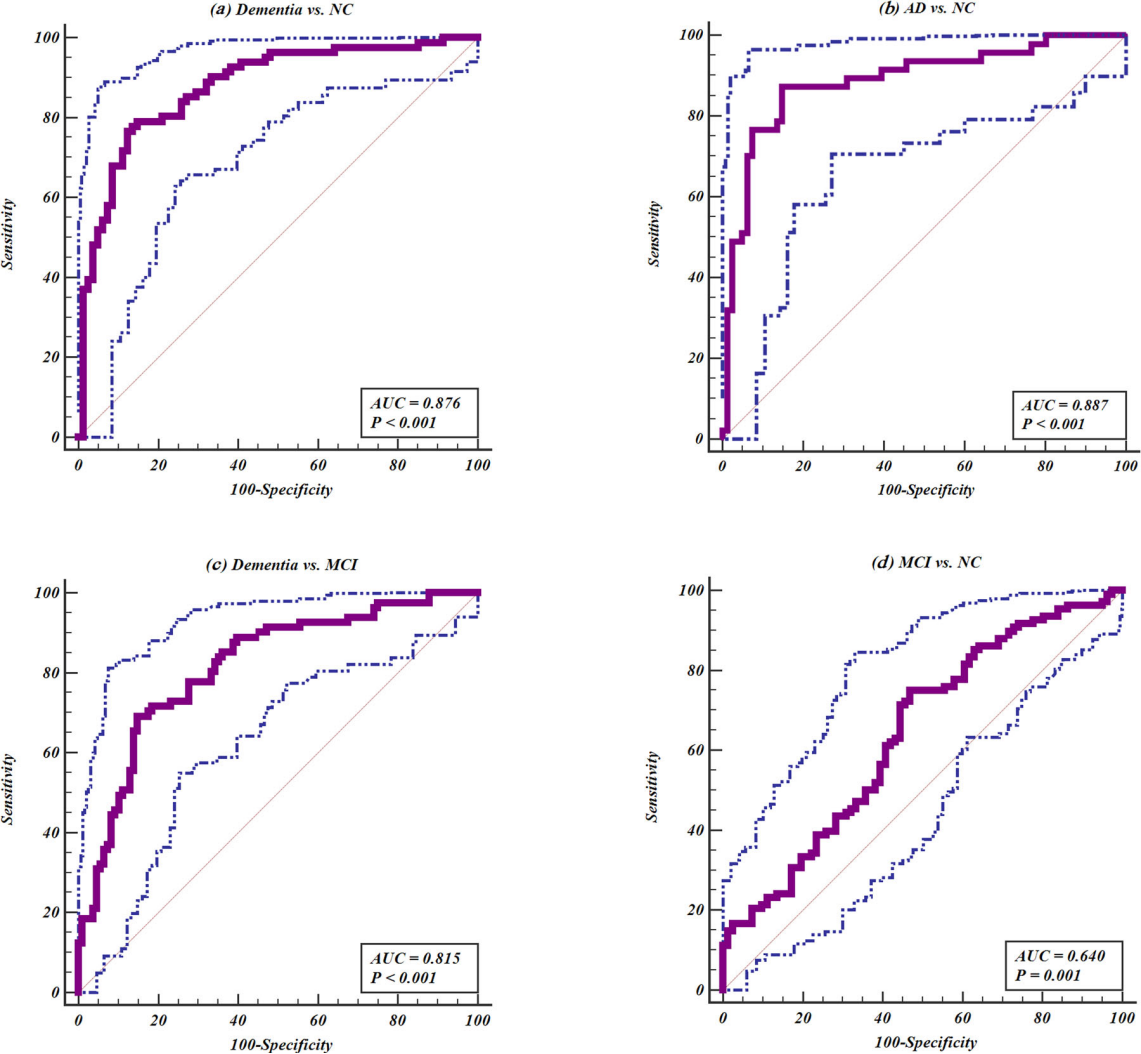
The ROC curves for the IgG *N*-glycans (GP8, GP9, and GP14) as diagnostic biomarkers by 5-fold cross-validation using the random forest classifier. The ROC curves are represented by purple color lines, while 95% confidence intervals (CIs) are represented by blue dotted lines. **a** Dementia vs. NC, **b** AD vs. NC, **c** dementia vs. MCI, and **d** MCI vs. NC. NC normal cognitive functioning, MCI mild cognitive impairment, AD Alzheimer’s disease.

As shown in Fig. 3, the significant correlations among glycans indicated that multicollinearity might exist (*p* < 0.05). GP8, GP9, and GP14 were selected by intersection of dimension reduction methods [the Ridge and Stepwise (including the direction of Forward and Backward) based on logistic regression as well as Lasso regression], and thus were identified as a biomarker panel for the diagnosis of dementia (Supplementary Table 7). Figure 4 showed the distribution of these three glycans for dementia, MCI, and NC groups. Models were trained and evaluated using 5-fold cross-validation in which random forest classifier was applied. The AUC value of the model including GP8, GP9, and GP14 was determined to distinguish dementia from NC groups as 0.876 (95% confidence interval [CI]: 0.815–0.923). Furthermore, this model was also able to distinguish AD from NC groups with an AUC of 0.887 (95% CI: 0.819–0.936), to distinguish dementia from MCI groups with an AUC of 0.815 (95% CI: 0.752–0.868), to distinguish MCI from NC groups with an AUC of 0.640 (95% CI: 0.568–0.709), respectively (Fig. 5). This model was of high accuracy to predict by performing the random forest classifier for different train datasets [normalized mean square error (NMSE) = 1.131, mean squared error (MSE) = 0.124, and mean absolute error (MAE) = 0.264 for dementia vs. NC; NMSE = 0.976, MSE = 0.075, and MAE = 0.218 for AD vs. NC; NMSE = 3.373, MSE = 0.224, and MAE = 0.401 for dementia vs. MCI; NMSE = 7.322, MSE = 0.234, and MAE = 0.445 for MCI vs. NC] (Supplementary Table 8). In addition, the panels of biomarkers were selected by intersection of three methods to reduce dimension for AD vs. NC, dementia vs. MCI, MCI vs. NC, and AD vs. MCI, respectively (Supplementary Tables 9–12). The AUCs showed high accuracy for diagnostic models in every two groups (Supplementary Table 13 and Supplementary Fig. 2).

### Correlation between IgG glycosylation and inflammation factors

The spearman correlation coefficients between *N*-glycans and inflammation factors were shown in Table 4. The anti-inflammatory (IL-4 and IL-10) state was positively correlated with GP8, GP10, and GP14 (*p* < 0.05). The proinflammation (CRP, TNF-α, and IFN-γ) factors were positively correlated with GP8 and GP10, whereas negatively correlated with GP16 (*p* < 0.05).

**Table 4:** Correlation coefficient (*r*_*s*_) between normalized initial glycans and inflammation factors.

Glycans	Anti-inflammatory factors		Proinflammatory factors					Inflammation
	IL-4	IL-10	CRP	TNF-α	IFN-γ	IL-1β	IL-6	
GP1	−0.050	0.003	0.050	0.007	−0.009	−0.006	0.025	/
GP2	−0.053	−0.026	0.069	0.069	−0.055	0.007	−0.058	/
GP4	−0.080	−0.050	0.017	0.046	0.011	0.088	−0.005	/
GP5	0.004	−0.056	0.073	0.053	−0.017	0.053	−0.013	/
GP6	−0.070	0.018	−0.052	0.043	0.040	−0.036	0.066	/
GP7	−0.007	−0.017	0.016	0.016	−0.042	−0.007	−0.072	/
GP8	−0.008	0.163*	0.168**	0.087	0.138*	0.035	0.042	+$
GP9	0.046	0.036	−0.023	−0.009	0.005	0.041	−0.040	/
GP10	−0.049	0.133*	0.137*	0.092	0.097	0.014	0.063	+$
GP11	0.038	−0.061	−0.010	−0.031	−0.047	−0.012	−0.015	/
GP12	−0.023	0.011	−0.005	0.046	−0.029	0.024	−0.071	/
GP13	0.045	−0.014	−0.012	−0.042	−0.119	−0.051	−0.063	/
GP14	0.135*	0.055	−0.060	−0.025	0.034	−0.051	0.065	+$
GP15	−0.021	−0.013	−0.016	0.013	−0.064	−0.003	−0.024	/
GP16	0.068	−0.123	−0.069	−0.133*	−0.092	−0.110	−0.028	−$
GP17	−0.057	0.002	0.011	−0.076	−0.072	−0.015	−0.092	/
GP18	0.050	−0.042	−0.102	−0.091	−0.048	−0.103	−0.001	/
GP19	−0.014	−0.060	−0.056	−0.063	−0.099	−0.065	−0.082	/
GP21	−0.033	−0.016	0.008	−0.013	0.017	0.008	−0.050	/
GP22	0.006	−0.053	0.000	−0.038	−0.070	0.005	−0.065	/
GP23	−0.004	−0.029	0.008	−0.074	−0.119	−0.009	−0.112	/
GP24	−0.019	−0.045	−0.064	−0.059	−0.009	−0.068	−0.047	/

*IL-4* interleukin-4, *IL-10* interleukin-10, *CRP* C-reactive protein, *TNF-α* tumor necrosis factor-alpha, *IFN-γ* interferon-gamma, *IL-1β* interleukin-1 beta, *IL-6* interleukin-6.

*Correlation is significant at the 0.05 level.

**Correlation is significant at the 0.01 level.

^$^There is at least one significant inflammation factor.

^/^The association between glycans and inflammation remains unclear.

^+^The association between glycans and inflammation is positive.

^−^The association between glycans and inflammation is negative.

## Discussion

In this study, we systematically investigated IgG *N*-glycan profiles in a case–control study including 81 dementia, 108 MCI, and 81 NC participants. To the best of our knowledge, the present study is the first study to assess the possible association between dementia and IgG glycosylation in Chinese Han population. The results demonstrated that 14 initial GPs reflecting the decreased of sialylation and core fucosylation, as well as the increased bisecting GlcNAc *N*-glycan structures were significantly associated with dementia after adjusting the effects of confounders. Diagnostic model including GP8, GP9, and GP14 was of promising capability to distinguish dementia from NC group with an AUC of 0.876 (95% CI: 0.815–0.923) and distinguish AD from NC group with an AUC of 0.887 (95% CI: 0.819–0.936) under 5-fold cross-validation random forest classifiers.

Dementia is a multifactorial disease driven by many diverse risk factors, including genetic variants, demographic risk factors (such as aging and gender), modifiable risk factors (central obesity, dyslipidemia, T2DM, hypertension, and ischemic stroke, etc.)^36^. Cumulative evidence indicates that variations in IgG Fc *N*-glycome play an important role in the anti-inflammatory or proinflammatory process^37^. Our previous studies have shown that the decreasing sialylation and the increasing bisecting GlcNAc are associated with risk factors of dementia, which are consistent with this study^28,30–34,38–40^. Therefore, aberrant glycosylation of IgG related to dementia implied a proinflammatory status in the participants with dementia in this study.

The associations between IgG Fc *N*-glycans and neurodegenerative diseases have been observed in previous studies^35,41–43^. Gizaw et al found that the fucose and bisecting-GlcNAc structures were significantly increased while disialylated *N*-glycans were decreased in AD patients’ serum when compared with the normal control group^41^. Maguire et al found that a significant decrease in soluble sialyltransferase activity in serum was reported in a study comparing 12 AD patients with 12 age-matched controls^42^. Lundström et al reported an increased FA2 (named GP4 in the present study) and a reduced FA2G1 (GP8 or GP9), FA2G2 (GP14), FA2G2S1 (GP18) of IgG1 in 31 patients with AD compared to 23 age-matched controls in European ancestry^35^. In addition, Russell et al found that the presence of Parkinson’s disease pathology in Caucasian population was predominantly explained with a reduced relative abundance of GP5, GP17, and an increased relative abundance of GP8^43^. We also detected decreasing GP14 and GP18 for dementia patients in Chinese population in this study. However, other results are inconsistent with these studies. Glycans do not have a direct genetic template, thus glycan structures attached to proteins are determined by these complex dynamic interactions including genetic and epigenetic factors^44,45^. Therefore, the extensive differences in genetics or environmental exposures among these ethnic studies may partly explain the inconsistent associations between IgG Fc *N*-glycans and neurodegenerative diseases.

**Fig. 6:**
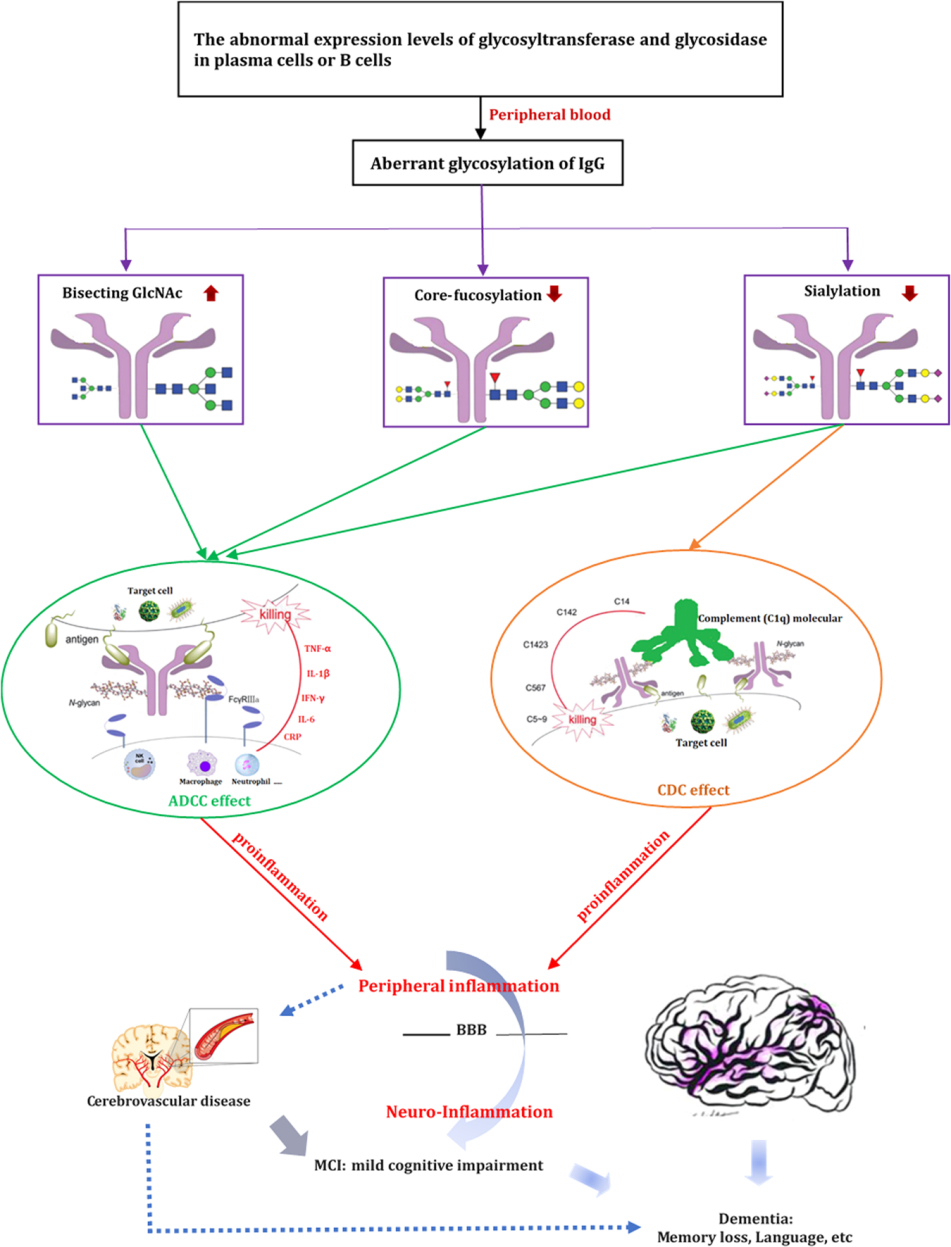
The proposed roles of IgG *N*-glycans in the pathogenesis of dementia. The genetic or environmental factors may influence the abnormal expression of glycosyltransferase and glycosidase in plasma cells or B cells. The decreasing sialylation and core fucosylation as well as increasing the bisecting GlcNAc contents within the Fc domain of IgG could activate its antibody-dependent cell mediated cytotoxicity (ADCC) by reducing inhibition to ligate Fcγ-RIIIa on natural killer (NK) cells, macrophage, and neutrophil which could release proinflammatory factors (such as IL-1β, IL-6, CRP, TNF-α, and IFN-γ). Decreased IgG Fc sialylation could reduce its complement-dependent cytotoxicity (CDC) activity via C1q binding, which could lead to increase activation of the lectin-initiated complement pathway. These changes of the IgG *N*-glycan may make them possible from anti-inflammatory to proinflammatory effects upon antigenic challenge. The central nervous system and peripheral immune system exchanged in bi-directional ways. Activated peripheral immune cells that can penetrate the blood brain barrier (BBB) may regulate cognitive impairment, which include inhibiting angiogenic, neurotrophic, and neuroprotective mechanisms. Consequently, distinct differences in IgG glycosylation pattern in MCI might play a cascading role in the progression of dementia. Besides, persistent inflammation related to atherosclerosis also might provide a possible explanation for the pathogenesis of cerebrovascular disease (such as hypertension in midlife and diabetes), which could increase risk of MCI and dementia. Arrows indicate a contributory effect.

IgG mediates pro- and anti-inflammatory activities mainly through the engagement of its constant region with distinct Fc receptors^46,47^. Studies have shown that the decreasing sialylation and core fucosylation as well as increasing the bisecting GlcNAc contents within the Fc domain of IgG could activate its antibody-dependent cell mediated cytotoxicity (ADCC) by reducing inhibition to ligate Fcγ-RIIIa on natural killer (NK) cells, macrophage, and neutrophil, which can release proinflammatory factors (such as IL-1β, IL-6, CRP, TNF-α, and IFN-γ)^17,48–51^. Thus, it may have the ability to fine-tune FcγRIII-mediated from anti-inflammatory to proinflammatory effects^20^. These alterations in the IgG *N*-glycan patterns may provide a switch from innate anti-inflammatory activity in the steady state to adaptive proinflammatory effects upon antigenic challenge^47^. Consistent with these, the inverse correlation between sialylated (Sia), and core fucosylated IgG glycoforms (Fuc and nFuc) and dementia in the present study was somewhat expected. Increased bisecting GlcNAc *N*-glycan structure (Bis and nBis) was observed to be related with the state of dementia in the present study. In addition, our findings that the level of IgG sialylation (Sia), and core fucosylation (Fuc and nFuc) decreased in MCI, while bisecting GlcNAc (Bis and nBis) increased in MCI compared with NC group, suggesting that aberrant glycosylation of IgG might contribute to pathogenesis of dementia developed from MCI stage. These were further validated by the significant differences of glycans between dementia and MCI in the present study. Consequently, it could be hypothesized that these distinct differences in IgG glycosylation pattern in MCI might play a cascading role in the progression of dementia (Fig. 6).

Our findings corroborate the results that peripheral inflammation correlates with dementia progress both prior to and following the onset of the disease^52–55^. Levels of proinflammation factors in serum were of significant differences among dementia, MCI and NC groups in the present study, which further validated possibly direct role of inflammation in the pathophysiology of MCI and dementia. According to the research findings, central nervous system and peripheral immune system exchanged in bi-directional ways^56^. Activated peripheral immune cells that can penetrate the blood brain barrier may regulate the pathogenesis of AD^57,58^. Inflammatory processes play an important role in cognitive impairment for the early phase of neurodegeneration which include inhibiting angiogenic, neurotrophic and neuroprotective mechanisms^59^. The high levels of proinflammatory factors (CRP, TNF-α, and IFN-γ) in the dementia group showed that inflammation might be one of the characteristics of dementia, which indicated that a chronic level of inflammation was a known risk factor for dementia^60^. We also found the anti-inflammatory (IL-10) state was positively correlated with GP8 and GP14, while the proinflammation (CRP and IFN-γ) factors were positively correlated with GP8. These findings indicated that significant changes of IgG glycosylation profiles might involve in the pathogenesis of dementia accompanying with inflammation response. The aberrant peripheral IgG glycome of individuals with dementia may reflect unknown modifications in the intermediate cellular environment or interactions of the biomolecules involved in B cell differentiation with the central nervous system at a known interaction site (Fig. 6). Besides, persistent inflammation related to atherosclerosis also might provide a possible explanation for the pathogenesis of cerebrovascular disease (such as hypertension and diabetes)^61,62^, implying an increased risk of MCI and dementia.

The study has several major strengths. First, to the best of our knowledge, this is the first study assessing IgG *N*-glycans levels in relation to the risk of dementia. Furthermore, we analyzed the association between IgG *N*-glycome and markers of inflammation including anti-inflammatory (IL-4 and IL-10) as well as proinflammatory factors (CRP, TNF-α, IFN-γ, IL-1β, and IL-6), contributing to explain the role of IgG glycosylation on dementia. However, This study has some limitations. First, because our study design was a case–control study, selection bias may still occur. In addition, it is very difficult to be warranted to infer causal relationships between the levels of IgG *N*-glycans and dementia. Second, all patients were on their dementia medications (donepezil, memantine, etc.), and it was not possible to distinguish the effects of dementia medications on the IgG glycome. Third, it is very hard to divide MCI into amnestic MCI (more likely due to AD) and non-amnestic groups based on available data in present study. Therefore, we failed to analyze and clarify why for some glycans there seem to be stepwise changes from NC to MCI to AD and for others MCI subjects have opposite trends from dementia and AD subjects. Lastly, because the sample size was not large enough, it was very hard to perform external cross-validation. Therefore, further external cross-validation validations for these novel biomarkers in cohort studies with large sample sizes and in multi-ethnic populations are also needed.

In conclusion, this study showed that individuals with dementia or on a pathway to dementia have an elevated proinflammatory activity in peripheral blood which manifests itself via the significant changes of IgG glycome profiles (reflecting the decreased of sialylation and core fucosylation, as well as the increased bisecting GlcNAc *N*-glycan structures). Aberrant IgG glycosylation might contribute to the pathogenesis of dementia because of the disequilibrium of anti- and proinflammatory status. Although it is yet to be determined whether the IgG glycosylation in blood plasma correlates with those in cerebrospinal fluid, it is evident that IgG *N*-glycans might contribute to the potential novel biomarkers for the neurodegenerative process risk assessment of dementia.

## Methods

### Participants

Participants were recruited from the Beijing Geriatric Hospital, the Emergency General Hospital, the Second Affiliated Hospital of Shandong First Medical University, the Tai’an City Central Hospital and the *Jidong* Oil-field Hospital of Chinese National Petroleum from May 2019 to January 2020. All participants were required to meet the following inclusion criteria: (1) Age 60 or above; (2) Chinese Han population; (3) signed informed consent prior to participation; and (4) blood sample available. Participants with the following diseases were excluded: rheumatoid arthritis, systemic lupus erythematosus and other rheumatoid immune disease. This study was approved by the Ethics Committee of the Capital Medical University, Beijing, China. The study was conducted according to the principles of the Declaration of Helsinki. Written informed consents were obtained from all participants before the study.

### Diagnostic criteria

Participants were classified into three general categories: normal cognitive functioning (NC), MCI and dementia. The Chinese version of the Montreal Cognitive Assessment Basic (MoCA-BC) was used as a quick evaluation scale to screen for MCI and distinguish MCI individuals from NC elderly adults^63^. The optimal cut-off scores for MCI detection were determined according to the education level. For individuals with 6 or fewer years of education, the cut-off score was set as less than or equal to 19. For individuals with 7–12 years of education were set as less than or equal to 22. For individuals with more than 12 years of education were set as less than or equal to 24. Diagnostic criteria for dementia were based on the fourth edition of the Diagnostic and Statistical Manual of Mental Disorders^64,65^. The diagnosis of AD was based on the National Institute of Neurological, Communicative Disorders and Stroke–Alzheimer Disease and Related Disorders Association (NINCDS-ADRDA)^66^.

### Collection of blood sample and clinical traits

After overnight fasting, two tubes of blood samples (2 mL) were collected in the morning by venipuncture. The tube without containing ethylene diamine tetraacetic acid (EDTA) was used to separate serum to measure the blood biochemistry indexes and inflammatory factors. The other tube of the blood sample in vacuum negative pressure tubes containing EDTA was centrifuged at 4000 rpm for 10 min to separate plasma. The plasma was used to measure IgG glycans. Collected blood samples were processed within 8 h and stored at −80 °C until further measurement.

Information on demographic (age, sex, ethnicity, and levels of education) and clinical history were collected by a questionnaire. Weight and height measurements were conducted when participants had removed their shoes and other heavy objects from their pockets. The body mass index (BMI) was calculated as weight in kilograms divided by height in meters squared. After the participants resting at least 10 min in a sitting position, SBP (mmHg) and DBP (mmHg) were measured twice on the right arm by trained nurses using a standard mercury sphygmomanometer^67^. According to the guideline for the prevention and control of dyslipidemia of adults in China, the participants were grouped into dyslipidemia with total cholesterol (TC) ≥ 6.2 mmol/L, or triglycerides (TG) ≥ 2.3 mmol/L, or high-density lipoprotein cholesterol (HDL-C) < 1.0 mmol/L or low-density lipoprotein cholesterol (LDL-C) ≥ 4.1 mmol/L^68^.

### Measurement of inflammatory factors and IgG *N*-glycans

The levels of inflammatory factors (IL-1β, IL-4, IL-6, IL-10, CRP, TNF-α, and IFN-γ) were measured using enzyme-linked immunosorbent assay (ELISA, R&D Systems, Bio-Techne China Co. Ltd., Beijing, China) according to the kits manufacturer’s instructions^69^. Take IL-6, for example. The standards, quality controls and serum samples were incubated in the micro-titration 96-well plate coated with polyclonal anti-human IL-6 antibody. Polyclonal anti-human IL-6 antibody labeled with horseradish peroxidase (HRP) was added to the wells and incubated with the immobilized antibody- IL-6 complex after a thorough wash. The HRP-conjugated antibody would react with the substrate and tetramethylbenzidine after another wash step. The reaction was stopped by acidic solution. Absorbance (optical density) was measured at 450 nm. The IL-6 concentration (mg/L) was interpolated from a four-parameter logistic 4-PL standard curve generated with ELISAcalc software. Each standard was estimated using the 4-PL curve-fit measurements.

The IgG was firstly isolated from human plasma as previously reported^70^. After washing and equilibrating protein G monolithic plates, 50 μL of plasma was diluted 10× with binding buffer (1× phosphate buffered saline, pH = 7.4) which was applied to the protein G plates, and then washed immediately. IgG was eluted with 1 mL of 0.1 M formic acid and neutralized with 1 M ammonium bicarbonate instantly. The following step was that IgG *N*-glycans were released and labeling was conducted. The released *N*-glycans were labeled with 2-aminobenzamide which was applied to make glycans visible by mixing with 2-aminobenzamide, dimethylsulfoxide, glacial acetic acid, and 2-picoline borane. In the end, IgG *N*-glycans were separated by hydrophilic interaction chromatography-UPLC into 24 IgG glycan peaks (GPs)^70^. The glycan structures of the most abundant glycans per peak were reported previously^71^.

All chromatograms were separated in the same manner into 24 peaks and the amount of glycans in each peak was expressed as a percentage of total integrated area as shown in Supplementary Table 1. The GP3 was excluded from all the calculations because it was eluted with contaminant in some samples that affected its value. In addition, the GP20 was also eliminated as its glycan structure has not been determined. The specific 54 derived traits representing the relative abundances of galactosylation, sialylation, bisecting *N*-acetylglucosamine (GlcNAc), and core fucosylation were calculated by the remaining 22 directly measured glycans^72^. In addition, Huff man et al. utilized another method to calculate overall aspects of derived traits^73^ (Supplementary Table 2). Normalization of UPLC data were detailed in previously published study^70^.

### Statistical analyses

Demographic and clinical characteristics were represented as mean ± standard deviation for continuous variables underlying the normal distribution, otherwise the median (*P*_25_ − *P*_75_) was used. The differences of continuous variables among three groups were tested by one-way analysis of variance or the Kruskal–Wallis test. Categorical variables were represented as n (%), and the differences among the three groups were tested by chi-square test or Fisher exact test.

Spearman correlations were used to calculate the correlation coefficient (*r*_*s*_) among IgG glycans as well as between IgG glycans and inflammatory factors. The multiple logistic regression analysis was performed to identify each of 24 initial glycans related to dementia after adjusting the effects of confounders (including BMI, levels of education, history of malignant tumor, habit of salt intake, ischemic stroke, diabetes, hypertension and dyslipidemia). For the multiple corrections, the FDR (false discovery rate) was used based on the Benjamini–Hochberg procedure (*q*)^74^. Then the Ridge and Stepwise (including the direction of Forward and Backward) based on logistic regression as well as Lasso regression were performed to reduce dimension for significant glycans^75–77^. The intersection of the above three methods was identified as a panel of biomarkers for the diagnosis of dementia. Model performance was evaluated using the 5-fold cross-validation^78^. Models were implemented using the random forest which was an ensemble or collection of multiple decision tree models. This panel of biomarkers simultaneously was evaluated performance for other comparison groups (AD vs. NC, dementia vs. MCI, and MCI vs. NC, respectively). Classifier accuracy was measured by using NMSE, MSE, MAE, and the AUC with the receiver operating characteristic (ROC) curve.

Data analysis was performed using SPSS Statistics version 25.0 for Windows (IBM Corp., Armonk, NY, USA) and R version 3.4.3 (R Core Team, 2017). All reported *p* values were two-tailed. The *q* value is used to represent the *p* value after correction for multiple testing and hence *q* value < 0.05 and *p* < 0.05 were considered statistically significant.

### Reporting summary

Further information on research design is available in the Nature Research Reporting Summary linked to this article.

## Supplementary information

Reporting Summary

Supplementary material

## Data Availability

The data that support the findings of this study are available from the corresponding author upon reasonable request.

## Supplementary information

**Supplementary information** The online version contains supplementary material available at 10.1038/s41514-021-00055-w.

## Code availability

The code generated in this study are available from the corresponding author upon reasonable request.

## References

[1] Prince, M. W. A., Guerchet, M., Ali, G. C., Wu, Y. T., Prina, M. *World Alzheimer Report 2015—The Global Impact of Dementia: An Analysis of Prevalence, Incidence, Cost and Trends*. (Alzheimer’s Disease International, London, 2015).

[2] Tangalos EG, Petersen RC (2018). Mild cognitive impairment in geriatrics. Clin. Geriatr. Med..

[3] Liuzzo G (2001). Atherosclerosis: an inflammatory disease. Rays.

[4] Livingston G (2017). Dementia prevention, intervention, and care. Lancet.

[5] Liu F, Wu S, Ren H, Gu J (2011). Klotho suppresses RIG-I-mediated senescence-associated inflammation. Nat. Cell Biol..

[6] Umeda-Kameyama Y, Akishita M (2016). Age and sex: risk factors for dementia. Brain Nerve.

[7] Helle (2003). Age-related inflammatory cytokines and disease - ScienceDirect. Immunol. Allergy Clin. North Am..

[8] King E (2018). Peripheral inflammation in prodromal Alzheimer’s and Lewy body dementias. J. Neurol. Neurosurg. Psychiatry.

[9] Koyama A (2013). The role of peripheral inflammatory markers in dementia and Alzheimer’s disease: a meta-analysis. J. Gerontol. Ser. A Biol. Sci. Med. Sci..

[10] Wood H (2018). Dementia: peripheral inflammation could be a prodromal indicator of dementia. Nat. Rev. Neurol..

[11] Darweesh SKL (2018). Inflammatory markers and the risk of dementia and Alzheimer’s disease: a meta-analysis. Alzheimer’s Dement..

[12] Shen XN (2019). Inflammatory markers in Alzheimer’s disease and mild cognitive impairment: a meta-analysis and systematic review of 170 studies. J. Neurol. Neurosurg. Psychiatry.

[13] Arnold JN, Wormald MR, Sim RB, Rudd PM, Dwek RA (2007). The impact of glycosylation on the biological function and structure of human immunoglobulins. Annu. Rev. Immunol..

[14] Molinari M (2007). N-glycan structure dictates extension of protein folding or onset of disposal. Nat. Chem. Biol..

[15] Ohtsubo K, Marth JD (2006). Glycosylation in cellular mechanisms of health and disease. Cell.

[16] Pinho SS, Reis CA (2015). Glycosylation in cancer: mechanisms and clinical implications. Nat. Rev. Cancer.

[17] Biermann MH (2016). Sweet but dangerous—the role of immunoglobulin G glycosylation in autoimmunity and inflammation. Lupus.

[18] Rudd PM, Elliott T, Cresswell P, Wilson IA, Dwek RA (2001). Glycosylation and the immune system. Science.

[19] Krištić J (2018). Profiling and genetic control of the murine immunoglobulin G glycome. Nat. Chem. Biol..

[20] Vidarsson G, Dekkers G, Rispens T (2014). IgG subclasses and allotypes: from structure to effector functions. Front. Immunol..

[21] Novokmet M (2014). Changes in IgG and total plasma protein glycomes in acute systemic inflammation. Sci. Rep..

[22] Vuckovic F (2015). Association of systemic lupus erythematosus with decreased immunosuppressive potential of the IgG glycome. Arthritis Rheumatol..

[23] Sebastian A (2016). Glycan biomarkers for rheumatoid arthritis and its remission status in Han Chinese patients. Omics.

[24] Barrios C (2016). Glycosylation profile of IgG in moderate kidney dysfunction. J. Am. Soc. Nephrol..

[25] Trbojevic Akmacic I (2015). Inflammatory bowel disease associates with proinflammatory potential of the immunoglobulin G glycome. Inflamm. Bowel Dis..

[26] de Jong SE (2016). IgG1 Fc N-glycan galactosylation as a biomarker for immune activation. Sci. Rep..

[27] Plomp R (2017). Subclass-specific IgG glycosylation is associated with markers of inflammation and metabolic health. Sci. Rep..

[28] Liu D (2018). Ischemic stroke is associated with the pro-inflammatory potential of N-glycosylated immunoglobulin G. J. Neuroinflamm..

[29] Liu JN (2018). The association between subclass-specific IgG Fc N-glycosylation profiles and hypertension in the Uygur, Kazak, Kirgiz, and Tajik populations. J. Hum. Hypertension.

[30] Liu D (2018). The changes of immunoglobulin G N-glycosylation in blood lipids and dyslipidaemia. J. Transl. Med..

[31] Yu X (2016). Profiling IgG N-glycans as potential biomarker of chronological and biological ages: a community-based study in a Han Chinese population. Medicine.

[32] Liu D (2019). The association between normal BMI with central adiposity and proinflammatory potential immunoglobulin G N-glycosylation. Diabetes Metab. Syndr. Obes..

[33] Liu J (2019). Glycomics for type 2 diabetes biomarker discovery: promise of immunoglobulin G subclass-specific fragment crystallizable N-glycosylation in the Uyghur population. Omics.

[34] Wang Y (2016). The association between glycosylation of immunoglobulin G and hypertension: a multiple ethnic cross-sectional study. Medicine.

[35] Lundström SL (2014). Blood plasma IgG Fc glycans are significantly altered in Alzheimer’s disease and progressive mild cognitive impairment. J. Alzheimers Dis..

[36] Hugo J, Ganguli M (2014). Dementia and cognitive impairment: epidemiology, diagnosis, and treatment. Clin. Geriatr. Med..

[37] Russell, A., Adua, E., Ugrina, I., Laws, S. & Wang, W. Unravelling immunoglobulin G Fc N-glycosylation: a dynamic marker potentiating predictive, preventive and personalised medicine. *Int. J. Mol. Sci.*10.3390/ijms19020390 (2018). 10.3390/ijms19020390 PMC5855612 29382131

[38] Li X (2019). Type 2 diabetes mellitus is associated with the immunoglobulin G N-glycome through putative proinflammatory mechanisms in an Australian population. Omics.

[39] Gao Q (2017). immunoglobulin G *N*-Glycans as potential postgenomic biomarkers for hypertension in the kazakh population. Omics.

[40] Ge S (2018). Type 2 diabetes mellitus: integrative analysis of multiomics data for biomarker discovery. Omics.

[41] Gizaw ST, Ohashi T, Tanaka M, Hinou H, Nishimura S (2016). Glycoblotting method allows for rapid and efficient glycome profiling of human Alzheimer’s disease brain, serum and cerebrospinal fluid towards potential biomarker discovery. Biochim. Biophys. Acta.

[42] Maguire TM (1994). A decrease in serum sialyltransferase levels in Alzheimer’s disease. Neurobiol. Aging.

[43] Russell AC (2017). The N-glycosylation of immunoglobulin G as a novel biomarker of Parkinson’s disease. Glycobiology.

[44] Kizuka, Y. & Taniguchi, N. Enzymes for N-glycan branching and their genetic and nongenetic regulation in cancer. *Biomolecules*10.3390/biom6020025 (2016). 10.3390/biom6020025 PMC4919920 27136596

[45] Menni C (2013). Glycosylation of immunoglobulin g: role of genetic and epigenetic influences. PloS ONE.

[46] Bond A, Alavi A, Axford JS, Youinou P, Hay FC (1996). The relationship between exposed galactose and N-acetylglucosamine residues on IgG in rheumatoid arthritis (RA), juvenile chronic arthritis (JCA) and Sjögren’s syndrome (SS). Clin. Exp. Immunol..

[47] Kaneko Y, Nimmerjahn F, Ravetch JV (2006). Anti-inflammatory activity of immunoglobulin G resulting from Fc sialylation. Science.

[48] Shields RL (2002). Lack of fucose on human IgG1 N-linked oligosaccharide improves binding to human Fcgamma RIII and antibody-dependent cellular toxicity. J. Biol. Chem..

[49] Shinkawa T (2003). The absence of fucose but not the presence of galactose or bisecting N-acetylglucosamine of human IgG1 complex-type oligosaccharides shows the critical role of enhancing antibody-dependent cellular cytotoxicity. J. Biol. Chem..

[50] Hodoniczky J, Zheng YZ, James DC (2005). Control of recombinant monoclonal antibody effector functions by Fc N-glycan remodeling in vitro. Biotechnol. Prog..

[51] Zou G (2011). Chemoenzymatic synthesis and Fcγ receptor binding of homogeneous glycoforms of antibody Fc domain. Presence of a bisecting sugar moiety enhances the affinity of Fc to FcγIIIa receptor. J. Am. Chem. Soc..

[52] Licastro F (2000). Increased plasma levels of interleukin-1, interleukin-6 and alpha-1-antichymotrypsin in patients with Alzheimer’s disease: peripheral inflammation or signals from the brain?. J. Neuroimmunol..

[53] De Luigi A (2002). Peripheral inflammatory response in Alzheimer’s disease and multiinfarct dementia. Neurobiol. Dis..

[54] Holmes C (2009). Systemic inflammation and disease progression in Alzheimer disease. Neurology.

[55] Heppner FL, Ransohoff RM, Becher B (2015). Immune attack: the role of inflammation in Alzheimer disease. Nat. Rev. Neurosci..

[56] Holmes C, Butchart J (2011). Systemic inflammation and Alzheimer’s disease. Biochem. Soc. Trans..

[57] Quan N, Banks WA (2007). Brain-immune communication pathways. Brain Behav. Immun..

[58] Takeda S, Sato N, Morishita R (2014). Systemic inflammation, blood-brain barrier vulnerability and cognitive/non-cognitive symptoms in Alzheimer disease: relevance to pathogenesis and therapy. Front. Aging Neurosci..

[59] Ott BR (2018). Blood-cerebrospinal fluid barrier gradients in mild cognitive impairment and alzheimer’s disease: relationship to inflammatory cytokines and chemokines. Front. Aging Neurosci..

[60] Akiyama H (2000). Inflammation and Alzheimer’s disease. Neurobiol. Aging.

[61] Agita A, Alsagaff MT (2017). Inflammation, immunity, and hypertension. Acta Med. Indones..

[62] Lontchi-Yimagou E, Sobngwi E, Matsha TE, Kengne AP (2013). Diabetes mellitus and inflammation. Curr. Diabetes Rep..

[63] Chen KL (2016). Validation of the Chinese Version of Montreal Cognitive Assessment Basic for screening mild cognitive impairment. J. Am. Geriatrics Soc..

[64] Do, L. L. T. N. *American Psychiatric Association Diagnostic and Statistical Manual of Mental Disorders (DSM-IV)*. (Springer, USA, 2011).

[65] Galvin JE, Sadowsky CH (2012). Practical guidelines for the recognition and diagnosis of dementia. J. Am. Board Fam. Med..

[66] McKhann G (1984). Clinical diagnosis of Alzheimer’s disease: report of the NINCDS-ADRDA Work Group under the auspices of Department of Health and Human Services Task Force on Alzheimer’s Disease. Neurology.

[67] Organization, W. H. A global brief on hypertension: silent killer, global public health crisis. *World Health Day* (2013).

[68] Wang S (2011). Prevalence and associated factors of dyslipidemia in the adult Chinese population. PloS ONE.

[69] Chang PH (2016). Pretreatment serum interleukin-1beta, interleukin-6, and tumor necrosis factor-alpha levels predict the progression of colorectal cancer. Cancer Med..

[70] Trbojevic-Akmacic I, Vilaj M, Lauc G (2016). High-throughput analysis of immunoglobulin G glycosylation. Expert Rev. Proteom..

[71] Lemmers RFH (2017). IgG glycan patterns are associated with type 2 diabetes in independent European populations. Biochim. Biophys. Acta Gen. Subj..

[72] Pucic M (2011). High throughput isolation and glycosylation analysis of IgG-variability and heritability of the IgG glycome in three isolated human populations. Mol. Cell. Proteom..

[73] Huffman JE (2014). Comparative performance of four methods for high-throughput glycosylation analysis of immunoglobulin G in genetic and epidemiological research. Mol. Cell. Proteom..

[74] Benjamini Y, Hochberg Y (1995). Controlling the false discovery rate: a practical and powerful Approach to multiple testing. J. R. Stat. Soc. Ser..

[75] Mcdonald GC (2009). Ridge regression. Wiley Interdiscip. Rev. Comput. Stat..

[76] Hans C (2009). Bayesian lasso regression. Biometrika.

[77] Shacham M, Brauner N (2014). Application of stepwise regression for dynamic parameter estimation. Comput. Chem. Eng..

[78] Grünauer A, Vincze M (2015). Using dimension reduction to improve the classification of high-dimensional. Data.

