# Supplementary material for: Association of dementia with immunoglobulin G N-glycans in a Chinese Han Population
Source: NPJ Aging Mech Dis. 2021 Feb 4;7:3. doi: 10.1038/s41514-021-00055-w (PMC7862610; doi:10.1038/s41514-021-00055-w)
Supplement: Supplementary file 1 — Reporting Summary [file 41514_2021_55_MOESM1_ESM.pdf]

nature research

Corresponding author(s): Youxin Wang and Wei Wang, NPJAMD-00285R1

Last updated by author(s): Dec 7, 2020

Reporting Summary

Nature Research wishes to improve the reproducibility of the work that we publish. This form provides structure for consistency and transparency in reporting. For further information on Nature Research policies, see our Editorial Policies and the Editorial Policy Checklist.

Statistics

For all statistical analyses, confirm that the following items are present in the figure legend, table legend, main text, or Methods section.

n/a	Confirmed	
☐	☒	The exact sample size (*n*) for each experimental group/condition, given as a discrete number and unit of measurement
☐	☒	A statement on whether measurements were taken from distinct samples or whether the same sample was measured repeatedly
☐	☒	The statistical test(s) used AND whether they are one- or two-sided<br>*Only common tests should be described solely by name; describe more complex techniques in the Methods section.*
☐	☒	A description of all covariates tested
☐	☒	A description of any assumptions or corrections, such as tests of normality and adjustment for multiple comparisons
☐	☒	A full description of the statistical parameters including central tendency (e.g. means) or other basic estimates (e.g. regression coefficient) AND variation (e.g. standard deviation) or associated estimates of uncertainty (e.g. confidence intervals)
☐	☒	For null hypothesis testing, the test statistic (e.g. *F*, *t*, *r*) with confidence intervals, effect sizes, degrees of freedom and *P* value noted<br>*Give P values as exact values whenever suitable.*
☒	☐	For Bayesian analysis, information on the choice of priors and Markov chain Monte Carlo settings
☒	☐	For hierarchical and complex designs, identification of the appropriate level for tests and full reporting of outcomes
☐	☒	Estimates of effect sizes (e.g. Cohen's *d*, Pearson's *r*), indicating how they were calculated

Our web collection on statistics for biologists contains articles on many of the points above.

Software and code

Policy information about availability of computer code

Data collection	Participants were recruited from the Beijing Geriatric Hospital, the Emergency General Hospital, the Second Affiliated Hospital of Shandong First Medical University, the Tai'an City Central Hospital and the Jidong Oil-field Hospital of Chinese National Petroleum from May 2019 to January 2020.
Data analysis	Data analysis was performed using SPSS Statistics version 25.0 for Windows (IBM Corp., Armonk, NY, USA) and R version 3.4.3 (R Core Team, 2017). All reported p values were two-tailed. The q value is used to represent the p value after correction for multiple testing and hence q value <0.05 and p <0.05 were considered statistically significant.

For manuscripts utilizing custom algorithms or software that are central to the research but not yet described in published literature, software must be made available to editors and reviewers. We strongly encourage code deposition in a community repository (e.g. GitHub). See the Nature Research guidelines for submitting code & software for further information.

Data

Policy information about availability of data

All manuscripts must include a data availability statement. This statement should provide the following information, where applicable:

- Accession codes, unique identifiers, or web links for publicly available datasets
- A list of figures that have associated raw data
- A description of any restrictions on data availability

The data that support the findings of this study are available from the corresponding author upon reasonable request.

Field-specific reporting

Please select the one below that is the best fit for your research. If you are not sure, read the appropriate sections before making your selection.

☒ Life sciences ☐ Behavioural & social sciences ☐ Ecological, evolutionary & environmental sciences

For a reference copy of the document with all sections, see nature.com/documents/nr-reporting-summary-flat.pdf

Life sciences study design

All studies must disclose on these points even when the disclosure is negative.

Sample size	In total, 81 partients with dementia, 81 age- and gender-matched participants with NC and 108 non-matched participants with MCI were included in the present study. The 81 dementia participants consisted of 47 (58.00%) AD, 18 (22.20%) vascular dementia (VaD), and 16 (19.80%) other type of dementia.
Data exclusions	Participants with the following diseases were excluded: rheumatoid arthritis, systemic lupus erythematosus and other rheumatoid immune disease.
Replication	Measurement of inflammatory factors and IgG N-glycans<br>The levels of inflammatory factors (IL-1β, IL-4, IL-6, IL-10, CRP, TNF-α, and IFN-γ) were measured using the ELISA kits (R&D Systems, Bio-Techne China Co. Ltd., Beijing, China).<br>The IgG was firstly isolated from human plasma as previously reported. After washing and equilibrating protein G monolithic plates, 50µL of plasma was diluted 10× with binding buffer (1× phosphate buffered saline, pH = 7.4) which was applied to the protein G plates, and then washed immediately. IgG was eluted with 1mL of 0.1M formic acid and neutralized with 1M ammonium bicarbonate instantly. The following step was that IgG N-glycans were released and labeling was conducted. The released N-glycans were labeled with 2-aminobenzamide which was applied to make glycans visible by mixing with 2-aminobenzamide, dimethylsulfoxide, glacial acetic acid, and 2-picoline borane. In the end, IgG N-glycans were separated by hydrophilic interaction chromatography-UPLC into 24 IgG glycan peaks (GPs). The glycan structures of the most abundant glycans per peak were reported previously.
Randomization	In total, 81 partients with dementia, 81 age- and gender-matched participants with NC and 108 non-matched participants with MCI were included in the present study.
Blinding	None

Reporting for specific materials, systems and methods

We require information from authors about some types of materials, experimental systems and methods used in many studies. Here, indicate whether each material, system or method listed is relevant to your study. If you are not sure if a list item applies to your research, read the appropriate section before selecting a response.

Materials & experimental systems

n/a	Involved in the study	
☐	☒	Antibodies
☒	☐	Eukaryotic cell lines
☒	☐	Palaeontology and archaeology
☒	☐	Animals and other organisms
☐	☒	Human research participants
☐	☒	Clinical data
☐	☒	Dual use research of concern

Methods

n/a	Involved in the study	
☒	☐	ChIP-seq
☒	☐	Flow cytometry
☒	☐	MRI-based neuroimaging

Antibodies

Antibodies used	Immunoglobulin G in human's plasm
Validation	Immunoglobulin G (IgG) plays an important role in the innate and adaptive immunity of the body. In terms of its extensive involvement in immunological processes, IgG is one of the mostly studied glycoproteins. The attachment of different glycans can significantly change conformation of the Fc with dramatic consequences for IgG effector functions. Glycosylation is a complex post-translational modification during gene expression involved in more than half of all mammalian proteins. Glycan binding in functional proteins has a crucial role in the biological processes of molecular recognition and adhesion, cell signaling or immunological response. Hence, molecular defects in glycan synthesis are recognized as direct causes of an increasing number of diseases.

Human research participants

Policy information about studies involving human research participants

Population characteristics	see above
Recruitment	All participants were required to meet the following inclusion criteria: (1) Age 60 or above; (2) Chinese Han population; (3) signed informed consent prior to participation; and (4) blood sample available.
Ethics oversight	This study was approved by the Ethics Committee of the Capital Medical University, Beijing, China. The study was conducted according to the principles of the Declaration of Helsinki.

Note that full information on the approval of the study protocol must also be provided in the manuscript.

Clinical data

Policy information about clinical studies

All manuscripts should comply with the ICMJE guidelines for publication of clinical research and a completed CONSORT checklist must be included with all submissions.

Clinical trial registration	None
Study protocol	None
Data collection	None
Outcomes	None

Dual use research of concern

Policy information about dual use research of concern

Hazards

Could the accidental, deliberate or reckless misuse of agents or technologies generated in the work, or the application of information presented in the manuscript, pose a threat to:

No	Yes	
☒	☐	Public health
☒	☐	National security
☒	☐	Crops and/or livestock
☒	☐	Ecosystems
☒	☐	Any other significant area

Experiments of concern

Does the work involve any of these experiments of concern:

No	Yes	
☒	☐	Demonstrate how to render a vaccine ineffective
☒	☐	Confer resistance to therapeutically useful antibiotics or antiviral agents
☒	☐	Enhance the virulence of a pathogen or render a nonpathogen virulent
☒	☐	Increase transmissibility of a pathogen
☒	☐	Alter the host range of a pathogen
☒	☐	Enable evasion of diagnostic/detection modalities
☒	☐	Enable the weaponization of a biological agent or toxin
☒	☐	Any other potentially harmful combination of experiments and agents
