# Supplementary material for: Association of dementia with immunoglobulin G N-glycans in a Chinese Han Population
Source: NPJ Aging Mech Dis. 2021 Feb 4;7:3. doi: 10.1038/s41514-021-00055-w (PMC7862610; doi:10.1038/s41514-021-00055-w)
Supplement: Supplementary file 2 — Supplementary material [file 41514_2021_55_MOESM2_ESM.pdf]

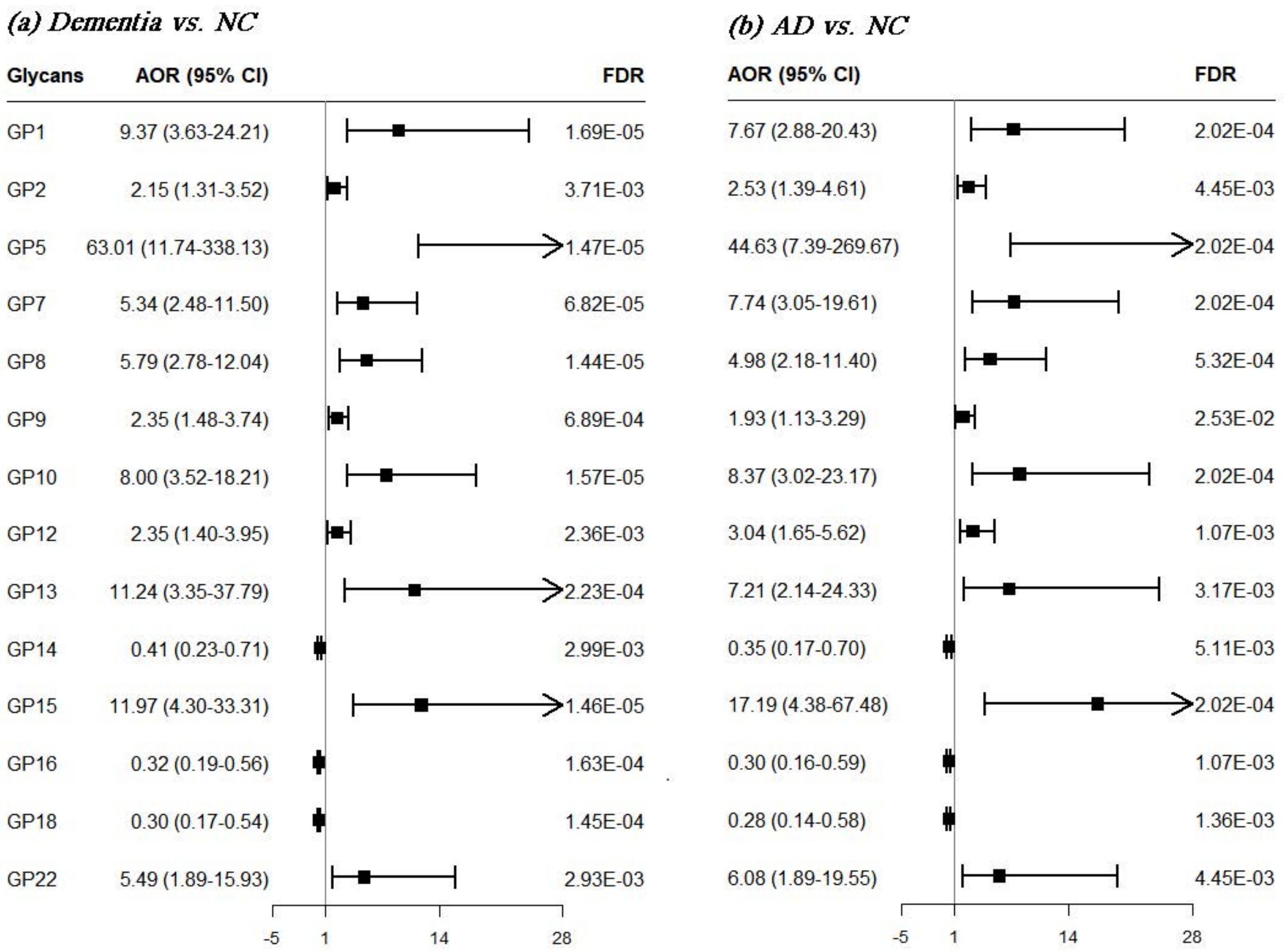

(a) Dementia vs. NC
Glycans
AOR (95% CI)
FDR
GP1 9.37 (3.63-24.21) 1.69E-05
GP2 2.15 (1.31-3.52) 3.71E-03
GP5 63.01 (11.74-338.13) 1.47E-05
GP7 5.34 (2.48-11.50) 6.82E-05
GP8 5.79 (2.78-12.04) 1.44E-05
GP9 2.35 (1.48-3.74) 6.89E-04
GP10 8.00 (3.52-18.21) 1.57E-05
GP12 2.35 (1.40-3.95) 2.36E-03
GP13 11.24 (3.35-37.79) 2.23E-04
GP14 0.41 (0.23-0.71) 2.99E-03
GP15 11.97 (4.30-33.31) 1.46E-05
GP16 0.32 (0.19-0.56) 1.63E-04
GP18 0.30 (0.17-0.54) 1.45E-04
GP22 5.49 (1.89-15.93) 2.93E-03
-5
1
14
28
(b) AD vs. NC
AOR (95% CI)
FDR
7.67 (2.88-20.43) 2.02E-04
2.53 (1.39-4.61) 4.45E-03
44.63 (7.39-269.67) 2.02E-04
7.74 (3.05-19.61) 2.02E-04
4.98 (2.18-11.40) 5.32E-04
1.93 (1.13-3.29) 2.53E-02
8.37 (3.02-23.17) 2.02E-04
3.04 (1.65-5.62) 1.07E-03
7.21 (2.14-24.33) 3.17E-03
0.35 (0.17-0.70) 5.11E-03
17.19 (4.38-67.48) 2.02E-04
0.30 (0.16-0.59) 1.07E-03
0.28 (0.14-0.58) 1.36E-03
6.08 (1.89-19.55) 4.45E-03
-5
1
14
28

Supplementary Figure 1 Forest plots of the associations of the IgG *N*-glycans with dementia and AD.

Forest plots (a, b) show the odds ratios (ORs, black solid square) with a horizontal lines representing 95% confidence intervals (CIs) for IgG *N*-glycans with dementia and AD. Each of statistically significant IgG *N*-glycans included study is represented by one row in the plots. The multiple logistic regression analysis was performed after adjusting for age, sex, BMI, levels of education, history of malignant tumor, habit of salt intake, ischemic stroke, diabetes, hypertension and dyslipidemia (adjusting for the above effects other than age and sex for dementia vs. NC). **(a)** Dementia *vs*. NC, **(b)** AD *vs*. NC.

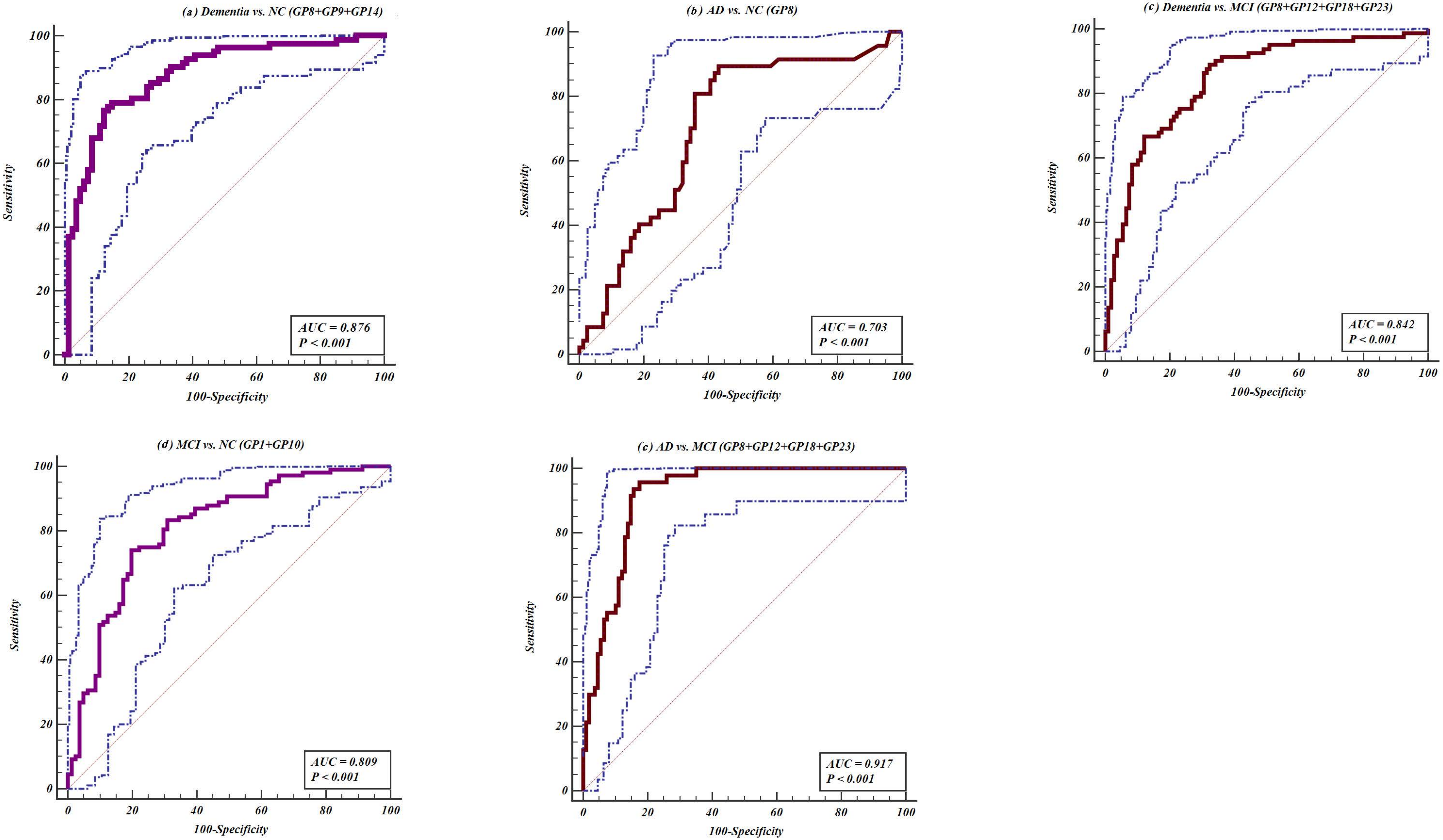
(a) Dementia vs. NC (GP8+GP9+GP14)
AUC = 0.876
P < 0.001
Sensitivity
100-Specificity
(b) AD vs. NC (GP8)
AUC = 0.703
P < 0.001
Sensitivity
100-Specificity
(c) Dementia vs. MCI (GP8+GP12+GP18+GP23)
AUC = 0.842
P < 0.001
Sensitivity
100-Specificity
(d) MCI vs. NC (GP1+GP10)
AUC = 0.809
P < 0.001
Sensitivity
100-Specificity
(e) AD vs. MCI (GP8+GP12+GP18+GP23)
AUC = 0.917
P < 0.001
Sensitivity
100-Specificity

Supplementary Figure 2 The ROC curves for the IgG *N*-glycans as diagnostic biomarkers by 5-fold cross-validation using the random forest classifier for every groups.

The ROC curves are represented by purple color lines, while 95% confidence intervals (CIs) are represented by blue dotted lines. **(a)** Dementia *vs*. NC (GP8+GP9+GP14), **(b)** AD *vs*. NC (GP8), **(c)** Dementia *vs*. MCI (GP8+GP12+GP18+GP23), **(d)** MCI *vs*. NC (GP1+GP10) and **(e)** AD *vs*. MCI (GP8+GP12+GP18+GP23).

NC: normal cognitive functioning; MCI: mild cognitive impairment; AD: Alzheimer's disease.

Supplementary Table 1 Description of the IgG glycome

Glycan peak	Structures of glycans	Peak composition	Glycan peak	Structures of glycans	Peak composition
GP1	FA1	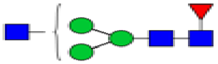	GP13	A2BG2	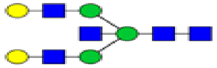
GP2	A2	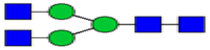	GP14	FA2G2	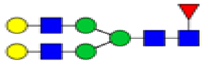
GP4	FA2	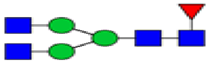	GP15	FA2BG2	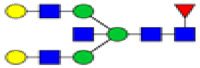
GP5	M5	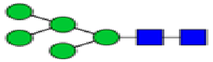	GP16	FA2G1S1	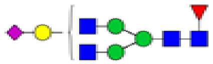
GP6	FA2B	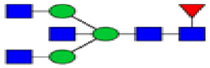	GP17	A2G2S1	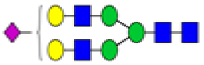
GP7	A2G1	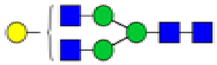	GP18	FA2G2S1	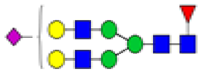
GP8	FA2[6]G1	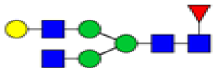	GP19	FA2BG2S1	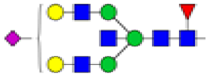
GP9	FA2[3]G1	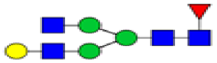	GP21	A2G2S2	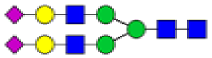
GP10	FA2[6]BG1	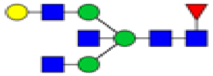	GP22	A2BG2S2	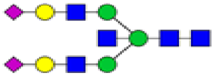

GP11	FA2[3]BG1	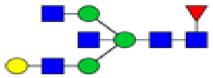	GP23	FA2G2S2	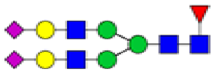
GP12	A2G2	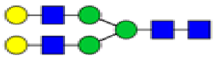	GP24	FA2BG2S2	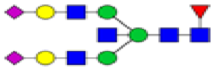

F represents a core fucose a-3 to -6 linked to the inner GlcNAcs; Ax, number (x) of GlcNAc (antenna) linked to the inner trimannose residues; Gx, number (x) of galactose on antenna-linked GlcNAc; Sx, number (x) of sialic acid residue on galactose; Mx, number (x) of mannose linked to core GlcNAcs; and B, bisecting GlcNAc linked to core mannose. Blue squares, bisecting GlcNAc; green circles, mannose; red triangles, fucose; yellow circles, galactose; purple diamonds, sialic acid. GP, glycan peak; GlcNAc, *N*-acetylglucosamine.

Supplementary Table 2 Index significance and computational method for derived glycans

Derived glycans	Index significance	Computational method
**Sialylation**		
FGS / (FG+FGS)	The percentage of sialylation of fucosylated galactosylated structures without bisecting GlcNAc in total IgG glycans	SUM (GP16 + GP18 + GP23) / SUM (GP16 + GP18 + GP23 + GP8 + GP9 + GP14) * 100
FBGS / (FBG+FBGS)	The percentage of sialylation of fucosylated galactosylated structures with bisecting GlcNAc in total IgG glycans	SUM (GP19 + GP24) / SUM (GP19 + GP24 + GP10 + GP11 + GP15) * 100
FGS / (F+FG+FGS)	The percentage of sialylation of all fucosylated structures without bisecting GlcNAc in total IgG glycans	SUM (GP16 + GP18 + GP23) / SUM (GP16 + GP18 + GP23 + GP4 + GP8 + GP9 + GP14) * 100
FBGS / (FB+FBG+FBGS)	The percentage of sialylation of all fucosylated structures with bisecting GlcNAc in total IgG glycans	SUM (GP19 + GP24) / SUM (GP19 + GP24 + GP6 + GP10 + GP11 + GP15) * 100
FG1S1 / (FG1+FG1S1)	The percentage of monosialylation of fucosylated monogalactosylated structures without bisecting GlcNAc in total IgG glycans	GP16 / SUM (GP16 + GP8 + GP9) * 100
FG2S1 / (FG2+FG2S1+FG2S2)	The percentage of monosialylation of fucosylated digalactosylated structures without bisecting GlcNAc in total IgG glycans	GP18 / SUM (GP18 + GP14 + GP23) * 100
FG2S2 / (FG2+FG2S1+FG2S2)	The percentage of disialylation of fucosylated digalactosylated structures without bisecting GlcNAc in total IgG glycans	GP23 / SUM (GP23 + GP14 + GP18) * 100
FBG2S1 / (FBG2+FBG2S1+FBG2S2)	The percentage of monosialylation of fucosylated digalactosylated structures with bisecting GlcNAc in total IgG glycans	GP19 / SUM (GP19 + GP15 + GP24) * 100

FBG2S2 / (FBG2+FBG2S1+FBG2S2)	The percentage of disialylation of fucosylated digalactosylated structures with bisecting GlcNAc in total IgG glycans	GP24 / SUM (GP24 + GP15 + GP19) * 100
$F^{total}S1$ / $F^{total}S2$	Ratio of all fucosylated monosialylated and disialylated structures (+/- bisecting GlyNAc) in total IgG glycans	SUM (GP16 + GP18 + GP19) / SUM (GP23 + GP24)
FS1 / FS2	Ratio of fucosylated monosialylated and disialylated structures (without bisecting GlcNAc) in total IgG glycans	SUM (GP16 + GP18) / GP23
FBS1 / FBS2	Ratio of fucosylated monosialylated and disialylated structures (with bisecting GlcNAc) in total IgG glycans	GP19 / GP24
FBS^{total} / FS^{total}	Ratio of all fucosylated sialylated structures with and without bisecting GlcNAc in total IgG glycans	SUM (GP19 + GP24) / SUM (GP16 + GP18 + GP23)
FBS1 / FS1	Ratio of fucosylated monosialylated structures with and without bisecting GlcNAc in total IgG glycans	GP19 / SUM (GP16 + GP18)
FBS1 / (FS1+FBS1)	The incidence of bisecting GlcNAc in all fucosylated monosialylated structures in total IgG glycans in total IgG glycans	GP19 / SUM (GP16 + GP18 + GP19)
FBS2 / FS2	Ratio of fucosylated disialylated structures with and without bisecting GlcNAc in total IgG glycans	GP24 / GP23
FBS2 / (FS2+FBS2)	The incidence of bisecting GlcNAc in all fucosylated disialylated structures in total IgG glycans	GP24 / SUM (GP23 + GP24)

Fuc	Core fucosylation	GP1+GP4+GP6+GP8+GP9+GP10+GP11+GP14+GP15+GP16+GP18+GP19+GP23+GP24
Bis	Bisecting *N*-acetylglucosamine	GP6+GP10+GP11+GP13+GP15+GP19+GP22+GP24
Sal	Sialylation	GP16+GP17+GP18+GP19+GP21+GP22+GP23+GP24
Sal-1	Monosialylation	GP16+GP17+GP18+GP19
Sal-2	Disialylation	GP21+GP22+GP23+GP24
**Neutral glycans**		
$GP1^n$	The percentage of FA1 glycan in total neutral IgG glycans (GP^n)	GP1 / GP^n* 100
$GP2^n$	The percentage of A2 glycan in total neutral IgG glycans (GP^n)	GP2 / GP^n* 100
$GP4^n$	The percentage of FA2 glycan in total neutral IgG glycans (GP^n)	GP4 / GP^n* 100
$GP5^n$	The percentage of M5 glycan in total neutral IgG glycans (GP^n)	GP5 / GP^n* 100
$GP6^n$	The percentage of FA2B glycan in total neutral IgG glycans (GP^n)	GP6 / GP^n* 100
$GP7^n$	The percentage of A2G1 glycan in total neutral IgG glycans (GP^n)	GP7 / GP^n* 100
$GP8^n$	The percentage of FA2[6]G1 glycan in total neutral IgG glycans (GP^n)	GP8 / GP^n* 100

$GP9^n$	The percentage of FA2[3]G1 glycan in total neutral IgG glycans (GP^n)	GP9 / GP^n* 100
$GP10^n$	The percentage of FA2[6]BG1 glycan in total neutral IgG glycans (GP^n)	GP10 / GP^n* 100
$GP11^n$	The percentage of FA2[3]BG1 glycan in total neutral IgG glycans (GP^n)	GP11 / GP^n* 100
$GP12^n$	The percentage of A2G2 glycan in total neutral IgG glycans (GP^n)	GP12 / GP^n* 100
$GP13^n$	The percentage of A2BG2 glycan in total neutral IgG glycans (GP^n)	GP13 / GP^n* 100
$GP14^n$	The percentage of FA2G2 glycan in total neutral IgG glycans (GP^n)	GP14 / GP^n* 100
$GP15^n$	The percentage of FA2BG2 glycan in total neutral IgG glycans (GP^n)	GP15 / GP^n* 100
**Neutral derived glycans**		
Gal	Galactosylation	GP7+GP8+GP9+GP10+GP11+GP12 +GP13+GP14+GP15+GP16+GP17+ GP18+GP19+GP21+GP22+GP23+G P24
Gal-1	Monogalactosylation	GP7+GP8+GP9+GP10+GP11+GP16
Gal-2	Digalactosylation	GP12+GP13+GP14+GP15+GP17+G P18+GP19+GP21+GP22+GP23+GP2 4
$G0^n$	The percentage of agalactosylated structures in total neutral IgG glycans	SUM ($GP1^n$: $GP4^n$ + $GP6^n$)

$G1^n$	The percentage of monogalactosylated structures in total neutral IgG glycans	SUM ($GP7^n$: $GP11^n$)
$G2^n$	The percentage of digalactosylated structures in total neutral IgG glycans	SUM ($GP12^n$: $GP15^n$)
nGal	The percentage of galactosylated structures in total neutral IgG glycans	(GP7+GP8+GP9+GP10+GP11+GP12+GP13+GP14+GP15)/GPn *100
$F^{n\ total}$	The sum of all fucosylated structures (+/- bisecting GlcNAc) in total neutral IgG glycans	SUM ($GP1^n$+ $GP4^n$+ $GP6^n$+ $GP8^n$+ $GP9^n$+ $GP10^n$+ $GP11^n$+ $GP14^n$+ $GP15^n$)
$FG0^{n\ total}$ / $G0^n$	The percentage of fucosylation of agalactosylated structures in total neutral IgG glycans	SUM ($GP1^n$+ $GP4^n$+ $GP6^n$) / $G0^n$ * 100
$FG1^{n\ total}$ / $G1^n$	The percentage of fucosylation of monogalactosylated structures in total neutral IgG glycans	SUM ($GP8^n$+ $GP9^n$+ $GP10^n$+ $GP11^n$) / $G1^n$ * 100
$FG2^{n\ total}$ / $G2^n$	The percentage of fucosylation of digalactosylated structures in total neutral IgG glycans	SUM ($GP14^n$+ GP15) / $G2^n$ * 100
F^n	The sum of fucosylated structures (without bisecting GlcNAc) in total neutral IgG glycans	SUM ($GP1^n$+ $GP4^n$+ $GP8^n$+ $GP9^n$+ $GP14^n$)
$FG0^n$ / $G0^n$	The percentage of fucosylation of agalactosylated structures (without bisecting GlcNAc) in total neutral IgG glycans	SUM ($GP1^n$+ $GP4^n$) / $G0^n$ * 100
$FG1^n$ / $G1^n$	The percentage of fucosylation of monogalactosylated structures (without bisecting GlcNAc) in total neutral IgG glycans	SUM ($GP8^n$+ $GP9^n$) / $G1^n$ * 100

$FG2^n$ / $G2^n$	The percentage of fucosylation of digalactosylated structures (without bisecting GlcNAc) in total neutral IgG glycans	$GP14^n$ / $G2^n$ * 100
FB^n	The sum of fucosylated structures (with bisecting GlcNAc) in total neutral IgG glycans	SUM ($GP6^n$ + $GP10^n$ + $GP11^n$ + $GP15^n$)
$FBG0^n$ / $G0^n$	The percentage of fucosylation of agalactosylated structures (with bisecting GlcNAc) in total neutral IgG glycans	$GP6^n$/ $G0^n$ * 100
$FBG1^n$ / $G1^n$	The percentage of fucosylation of monogalactosylated structures (with bisecting GlcNAc) in total neutral IgG glycans	SUM ($GP10^n$ + $GP11^n$) / $G1^n$ * 100
$FBG2^n$ / $G2^n$	The percentage of fucosylation of digalactosylated structures (with bisecting GlcNAc) in total neutral IgG glycans	$GP15^n$ / $G2^n$ * 100
FB^n / F^n	Ratio of fucosylated structures with and without bisecting GlcNAc in total neutral IgG glycans	FB^n / F^n * 100
FB^n / $F^{n\ total}$	The incidence of bisecting GlcNAc in all fucosylated structures in total neutral IgG glycans	FB^n / F^n total * 100
F^n / (B^n + FB^n)	Ratio of fucosylated non-bisecting GlcNAc structures and all structures with bisecting GlcNAc in total neutral IgG glycans	F^n / ($GP13^n$ + FB^n)
B^n / (F^n + FB^n)	Ratio of structures with bisecting GlcNAc and all fucosylated structures (+/- bisecting GlcNAc) in total neutral IgG glycans	$GP13^n$ / (F^n+ FB^n) * 1000
$FBG2^n$ / $FG2^n$	Ratio of fucosylated digalactosylated structures with and without bisecting GlcNAc in total neutral IgG glycans	$GP15^n$ / $GP14^n$

$FBG2^n$ / ($FG2^n$ + $FBG2^n$)	The incidence of bisecting GlcNAc in all fucosylated digalactosylated structures in total neutral IgG glycans	$GP15^n$ / ($GP14^n$ + $GP15^n$) * 100
$FG2^n$ / ($BG2^n$ + $FBG2^n$)	Ratio of fucosylated digalactosylated non-bisecting GlcNAc structures and all digalactosylated structures with bisecting GlcNAc in total neutral IgG glycans	$GP14^n$ / ($GP13^n$ + $GP15^n$)
$BG2^n$ / ($FG2^n$ + $FBG2^n$)	Ratio of digalactosylated structures with bisecting GlcNAc and all fucosylated digalactosylated structures (+/- bisecting GlcNAc) in total neutral IgG glycans	$GP13^n$ / ($GP14^n$ + $GP15^n$) * 1000
nFuc	The percentage of core fucosylated structures in total neutral IgG glycans	(GP1+GP4+GP6+GP8+GP9+GP10+ GP11+GP14+GP15)/GP^n*100
nBis	The percentage of Bisecting *N*-acetylglucosamine structures in total neutral IgG glycans	GP6+GP10+GP11+GP13+GP15)/GP^n* 100

Supplementary Table 3 Normality test of the IgG glycome

Glycan peak	*p* value* NC (*n*=81)	*p* value* MCI (*n*=108)	*p* value* Dementia (*n*=81)
GP1	<0.001	<0.001	0.001
GP2	<0.001	<0.001	<0.001
GP4	0.200	0.200	0.191
GP5	<0.001	<0.001	<0.001
GP6	0.200	0.002	0.200
GP7	<0.001	<0.001	<0.001
GP8	0.200	0.029	0.200
GP9	0.012	0.069	0.200
GP10	<0.001	<0.001	0.200
GP11	<0.001	<0.001	<0.001
GP12	<0.001	<0.001	<0.001
GP13	<0.001	<0.001	<0.001
GP14	0.200	0.200	0.200
GP15	<0.001	<0.001	0.168
GP16	0.200	0.200	0.200
GP17	0.200	<0.001	<0.001
GP18	0.200	0.011	0.200
GP19	<0.001	0.137	0.200
GP21	<0.001	<0.001	<0.001
GP22	<0.001	<0.001	<0.001
GP23	<0.001	<0.001	0.200
GP24	<0.001	<0.001	<0.001

$p < 0.05$ was considered statistically significant.
NC: normal cognitive functioning; MCI: mild cognitive impairment. GP, glycan peak.
Normality distributions of glycans were tested by the Kolmogorov-Smirnov tests.

Supplementary Table 4 The levles of derived glycans from NC, MCI and dementia patients

Derived glycans	NC (n=81)	MCI (n=108)	Dementia (n=81)	p value*
	Median (P_{25}-P_{75})	Median (P_{25}-P_{75})	Median (P_{25}-P_{75})	
**Sialylation**				
FGS/(FG+FGS)	34.56 (29.21-39.32) $^{\$\&}$	31.56 (29.08-37.52)	26.67 (23.66-30.30)	<0.001
FBGS/(FBG+FBGS)	72.50 (61.44-79.38) $^{\#\$\&}$	61.02 (48.24-71.81)	48.23 (37.69-59.29)	<0.001
FGS/(F+FG+FGS)	17.33 (14.53-21.86) $^{\$\&}$	15.80 (13.64-20.05)	13.75 (11.07-16.42)	<0.001
FBGS/(FB+FBG+FBGS)	27.54 (19.38-36.09) $^{\$}$	24.66 (17.98-32.30)	20.92 (16.27-25.57)	0.003
FG1S1/(FG1+FG1S1)	20.82 (15.51-26.47) $^{\$\&}$	18.54 (15.18-23.05)	13.72 (10.52-16.37)	<0.001
FG2S1/(FG2+FG2S1+FG2S2)	41.20 (36.53-44.76) $^{\$\&}$	40.84 (37.53-44.52)	37.70 (34.49-40.53)	<0.001
FG2S2/(FG2+FG2S1+FG2S2)	2.92 (1.72-3.73) $^{\$\&}$	2.47 (1.68-3.30)	3.98 (2.89-5.42)	<0.001
FBG2S1/(FBG2+FBG2S1+FBG2S2)	63.84 (57.33-69.00) $^{\$}$	61.85 (53.37-67.60)	57.14 (49.77-63.88)	0.002
FBG2S2/(FBG2+FBG2S1+FBG2S2)	30.94 (23.70-37.41) $^{\$}$	29.63 (21.61-36.40)	24.36 (19.78-33.09)	0.022
$F^{total}S1/F^{total}S2$	9.21 (7.59-13.16) $^{\$\&}$	10.19 (7.58-14.28)	7.80 (5.80-9.75)	<0.001
FS1/FS2	20.15 (14.84-29.07) $^{\$\&}$	24.36 (17.23-37.92)	12.87 (10.29-18.51)	<0.001
FBS1/FBS2	2.05 (1.56-2.72)	2.10 (1.54-3.05)	2.28 (1.60-3.15)	0.663
FBS^{total}/FS^{total}	0.18 (0.12-0.26) $^{\$\&}$	0.20 (0.15-0.27)	0.24 (0.18-0.29)	0.003
FBS1/FS1	0.13 (0.09-0.19) $^{\$}$	0.14 (0.10-0.20)	0.17 (0.12-0.20)	0.004
FBS1/(FS1+FBS1)	11.79 (7.84-15.82) $^{\$}$	12.45 (9.03-16.57)	14.66 (11.09-17.00)	0.004
FBS2/FS2	1.18 (0.81-2.10) $^{\&}$	1.56 (0.80-2.38)	0.94 (0.72-1.39)	0.003
FBS2/(FS2+FBS2)	54.15 (44.62-67.66) $^{\&}$	60.92 (44.39-70.37)	48.37 (41.74-58.11)	0.003
Fuc	97.65 (96.99-98.12) $^{\#\$}$	96.62 (95.25-97.30)	96.15 (93.57-97.26)	<0.001
Bis	10.62 (9.31-12.55) $^{\#\$}$	12.05 (10.28-14.35)	13.25 (11.18-14.83)	<0.001
Sia	18.78 (15.69-24.31) $^{\$\&}$	17.20 (14.67-20.81)	15.22 (12.82-18.34)	<0.001
Sia-1	16.63 (14.00-21.22) $^{\$\&}$	15.41 (13.21-18.63)	13.25 (10.74-15.16)	<0.001
Sia-2	2.10 (1.53-3.15)	1.77 (1.31-2.53)	2.10 (1.53-3.15)	0.050
**Neutral glycans**				
$GP1^n$	0.07 (0.03-0.25) $^{\#\$}$	0.46 (0.20-0.73)	0.38 (0.25-0.68)	<0.001
$GP2^n$	0.34 (0.13-0.54 $^{\#\$}$	0.47 (0.23-0.91)	0.55 (0.29-0.98)	0.002
$GP4^n$	53.23 (45.41-56.98) $^{\$}$	50.63 (45.44-57.45)	47.57 (42.38-54.57)	0.022
$GP5^n$	0.00 (0.00-0.02) $^{\#\$\&}$	0.07 (0.01-0.22)	0.24 (0.08-0.43)	<0.001
$GP6^n$	7.95 (6.20-9.60)	7.86 (6.49-9.25)	7.83 (6.45-9.05)	0.993
$GP7^n$	0.01 (0.00-0.06) $^{\#\$}$	0.17 (0.06-0.33)	0.17 (0.06-0.50)	<0.001
$GP8^n$	14.41 (12.76-16.10) $^{\$\&}$	15.15 (12.79-17.37)	17.58 (15.84-19.37)	<0.001
$GP9^n$	3.84 (2.10-5.58) $^{\wedge\$}$	4.31 (2.61-6.04)	5.14 (3.84-6.78)	0.001
$GP10^n$	0.91 (0.65-1.49) $^{\#\$\&}$	1.44 (0.69-2.45)	2.52 (1.46-3.50)	<0.001

$GP11^n$	0.11 (0.05-0.31) $^{\#\&}$	0.39 (0.16-0.74)	0.12 (0.05-0.35)	<0.001
$GP12^n$	0.14 (0.08-0.31) $^{\$\&}$	0.20 (0.09-0.38)	0.38 (0.20-0.73)	<0.001
$GP13^n$	0.17 (0.05-0.41) $^{\#\$}$	0.39 (0.19-0.94)	0.42 (0.29-0.95)	<0.001
$GP14^n$	17.47 (13.12-22.81) $^{\wedge\ \$}$	15.59 (10.18-21.23)	13.35 (9.66-17.90)	<0.001
$GP15^n$	0.13 (0.07-0.22) $^{\#\$\&}$	0.26 (0.14-0.53)	0.52 (0.31-0.84)	<0.001
**Galactosylation**				
Gal	51.08±10.42	49.95±9.38	50.09±10.39	0.718
Gal-1	20.10±3.25 $^{\#\$\&}$	21.58±3.58	24.33±3.55	<0.001
Gal-2	30.98±9.03 $^{\$}$	28.37±8.56	25.76±8.26	0.001
$G0^n$	62.27 (53.42-67.61)	61.38 (53.96-67.52)	57.82 (51.62-63.49)	0.077
$G1^n$	19.89 (17.42-22.93) $^{\wedge\$}$	22.11 (19.53-25.03)	26.23 (23.27-29.81)	<0.001
$G2^n$	17.84 (13.67-23.71) $^{\wedge\#\$\&}$	16.80 (11.26-22.58)	15.50 (11.39-20.40)	0.025
nGal	37.73 (32.29-46.58)	38.57 (32.44-46.04)	41.72 (36.02-48.16)	0.088
**Core fucosylation and bisecting *N*-acetylglucosamine**				
F^{ntotal}	99.20 (98.54-99.43) $^{\#\$}$	98.38 (97.65-98.96)	98.45 (96.67-99.03)	<0.001
$FG0^{ntotal}/G0^n$	99.48 (99.08-99.76) $^{\#\$}$	99.14 (98.56-99.59)	98.98 (98.26-99.47)	<0.001
$FG1^{ntotal}/G1^n$	99.93 (99.69-100.00) $^{\#\$}$	99.14 (98.63-99.73)	99.36 (98.07-99.78)	<0.001
$FG2^{ntotal}/G2^n$	98.04 (95.85-98.70) $^{\#\$\&}$	95.49 (91.96-97.26)	94.19 (90.87-96.24)	<0.001
F^n	89.43 (86.84-91.80) $^{\#\$}$	87.88 (85.40-89.94)	86.07 (83.83-89.10)	<0.001
$FG0^n/G0^n$	86.23 (82.65-88.68) $^{\wedge}$	85.99 (82.89-87.93)	85.20 (82.01-87.77)	0.063
$FG1^n/G1^n$	93.75 (91.01-95.60) $^{\#\$}$	90.49 (86.26-93.21)	88.08 (85.31-91.27)	<0.001
$FG2^n/G2^n$	96.95 (94.47-98.09) $^{\#\$\&}$	92.90 (89.60-95.85)	89.84 (85.57-93.34)	<0.001
FB^n	9.57 (7.68-11.71) $^{\wedge\ \$}$	9.97 (8.43-12.40)	11.20 (9.82-13.64)	0.001
$FBG0^n/G0^n$	13.39 (10.78-16.11)	13.03 (10.94-15.34)	13.99 (11.11-16.15)	0.295
$FBG1^n/G1^n$	6.07 (4.40-8.32) $^{\#\$}$	8.18 (5.86-12.23)	10.88 (7.52-13.01)	<0.001
$FBG2^n/G2^n$	0.75 (0.44-1.28) $^{\#\$\&}$	1.51 (0.70-3.56)	3.99 (2.33-5.51)	<0.001
FB^n/F^n	10.71 (8.35-13.61) $^{\$}$	11.26 (9.36-14.37)	12.99 (11.00-16.31)	<0.001
$FB^n/F^{n\ total}$	9.67 (7.70-11.98) $^{\$}$	10.12 (8.56-12.56)	11.50 (9.91-14.03)	<0.001
$F^n/(B^n + FB^n)$	9.15 (7.29-11.62) $^{\#\$}$	8.18 (6.60-9.71)	6.96 (5.92-8.69)	<0.001
$B^n/(F^n + FB^n)$	1.68 (0.52-4.20) $^{\#\$}$	3.91 (1.91-9.60)	4.23 (2.94-9.76)	<0.001
$FBG2^n/FG2^n$	0.01 (0.00-0.01) $^{\#\$\&}$	0.02 (0.01-0.04)	0.04 (0.03-0.06)	<0.001
$FBG2^n/(FG2^n + FBG2^n)$	0.76 (0.46-1.30) $^{\#\$\&}$	1.56 (0.72-3.65)	4.16 (2.53-6.02)	<0.001
$FG2^n/(BG2^n + FBG2^n)$	49.48 (25.65-98.00) $^{\#\$\&}$	17.98 (10.06-33.70)	12.49 (8.49-19.75)	<0.001
$BG2^n/(FG2^n + FBG2^n)$	10.03 (2.58-22.85) $^{\#\$}$	29.07 (11.50-67.86)	34.06 (21.40-49.39)	<0.001
nFuc	99.13 (98.53-99.43) $^{\#\$}$	98.31 (97.31-98.78)	98.15 (96.19-98.87)	<0.001
nBis	9.84 (7.90-11.82) $^{\#\$}$	10.67 (9.22-12.97)	12.26 (10.25-14.13)	<0.001

NC: normal cognitive functioning; MCI: mild cognitive impairment.

^ Analysis of Variance (ANOVA)
* $p < 0.05$ was considered statistically significant.
#$p < 0.017$, MCI group compared with NC group
$$p < 0.017$, Dementia group compared with NC group
&$p < 0.017$, Dementia group compared with MCI group

Supplementary Table 5 Associations of the derived traits

Derived traits	MCI *vs.* NC			Dementia *vs.* NC			Dementia *vs.* MCI		
	OR (95% CI)#	*p-adjusted*	*q**	OR(95% CI) #	*p-adjusted*	*q**	OR (95% CI) #	*p-adjusted*	*q**
FGS/(FG+FGS)	0.97 (0.69-1.38)	8.83E-01	8.83E-01	0.22 (0.12-0.43)	5.48E-06	2.33E-05	0.22 (0.10-0.45)	4.48E-05	4.57E-04
FBGS/(FBG+FBGS)	0.39 (0.24-0.62)	*6.38E-05*	*4.07E-04*	0.26 (0.15-0.44)	6.67E-07	8.50E-06	0.83 (0.52-1.32)	4.29E-01	7.06E-01
FGS/(F+FG+FGS)	0.65 (0.43-0.97)	*3.63E-02*	*5.98E-02*	0.43 (0.28-0.67)	1.80E-04	4.59E-04	0.42 (0.24-0.74)	2.82E-03	1.80E-02
FBGS/(FB+FBG+FBGS)	0.59 (0.39-0.90)	*1.52E-02*	*2.87E-02*	0.51 (0.32-0.81)	4.01E-03	6.60E-03	0.96 (0.59-1.57)	8.76E-01	9.63E-01
FG1S1/(FG1+FG1S1)	0.92 (0.65-1.30)	6.26E-01	6.51E-01	0.13 (0.06-0.30)	1.09E-06	1.10E-05	0.15 (0.06-0.35)	1.07E-05	2.96E-04
FG2S1/(FG2+FG2S1+FG2S2)	1.53 (1.00-2.33)	5.16E-02	7.74E-02	0.50 (0.27-0.94)	3.01E-02	4.27E-02	0.37 (0.21-0.67)	8.84E-04	7.51E-03
FG2S2/(FG2+FG2S1+FG2S2)	0.33 (0.12-0.93)	3.53E-02	5.98E-02	1.91 (0.84-4.35)	1.25E-01	1.52E-01	18.56 (5.03-68.47)	1.16E-05	2.96E-04
FBG2S1/(FBG2+FBG2S1+FBG2S2)	0.88 (0.61-1.28)	5.11E-01	5.61E-01	0.59 (0.39-0.91)	1.56E-02	2.41E-02	0.74 (0.50-1.10)	1.31E-01	2.99E-01
FBG2S2/(FBG2+FBG2S1+FBG2S2)	0.73 (0.50-1.07)	1.08E-01	1.45E-01	0.76 (0.50-1.15)	1.93E-01	2.29E-01	1.15 (0.78-1.70)	4.77E-01	7.08E-01
$F^{total}S1/F^{total}S2$	2.41 (1.10-5.28)	2.85E-02	5.01E-02	0.23 (0.06-0.85)	2.72E-02	3.96E-02	0.15 (0.03-0.64)	1.07E-02	5.43E-02
FS1/FS2	3.87 (1.32-11.37)	1.38E-02	2.71E-02	0.13 (0.03-0.63)	1.16E-02	1.85E-02	0.01 (0.00-0.10)	3.71E-05	4.57E-04
FBS1/FBS2	2.11 (0.88-5.04)	9.38E-02	1.29E-01	1.49 (0.20-11.33)	7.00E-01	7.28E-01	0.43 (0.19-0.96)	3.92E-02	1.18E-01
FBS^{total}/FS^{total}	0.89 (0.63-1.27)	5.22E-01	5.61E-01	1.33 (0.95-1.85)	9.72E-02	1.21E-01	2.25 (1.14-4.46)	2.00E-02	7.86E-02
FBS1/FS1	0.89 (0.63-1.24)	4.73E-01	5.61E-01	1.18 (0.89-1.57)	2.49E-01	2.76E-01	2.10 (1.02-4.33)	4.52E-02	1.28E-01
FBS1/(FS1+FBS1)	0.93 (0.67-1.31)	6.88E-01	7.02E-01	1.42 (0.99-2.03)	5.50E-02	7.38E-02	1.79 (1.05-3.06)	3.33E-02	1.06E-01
FBS2/FS2	1.14 (0.76-1.72)	5.25E-01	5.61E-01	0.96 (0.68-1.37)	8.38E-01	8.54E-01	0.86 (0.56-1.33)	5.05E-01	7.08E-01
FBS2/(FS2+FBS2)	1.20 (0.81-1.77)	3.71E-01	4.51E-01	0.81 (0.51-1.29)	3.78E-01	4.01E-01	0.71 (0.45-1.11)	1.29E-01	2.99E-01
Fuc	0.04 (0.01-0.15)	3.93E-06	1.54E-04	0.13 (0.05-0.37)	1.17E-04	3.32E-04	0.71 (0.44-1.15)	1.64E-01	3.35E-01
Bis	1.52 (0.99-2.35)	5.73E-02	8.35E-02	1.97 (1.25-3.11)	3.70E-03	6.29E-03	1.19 (0.75-1.87)	4.62E-01	7.08E-01
Sia	0.55 (0.36-0.83)	4.79E-03	1.36E-02	0.38 (0.24-0.61)	5.75E-05	1.83E-04	0.53 (0.32-0.90)	1.96E-02	7.86E-02
Sia-1	0.57 (0.38-0.86)	7.39E-03	1.79E-02	0.29 (0.17-0.49)	5.41E-06	2.33E-05	0.39 (0.22-0.69)	1.26E-03	9.20E-03
Sia-2	0.51 (0.31-0.85)	9.29E-03	2.15E-02	0.99 (0.71-1.38)	9.65E-01	9.65E-01	1.58 (0.97-2.56)	6.53E-02	1.75E-01
Gal	0.73 (0.52-1.04)	7.88E-02	1.09E-01	0.94 (0.63-1.39)	7.56E-01	7.56E-01	1.23 (0.84-1.81)	2.94E-01	4.58E-01
Gal-1	1.60 (1.12-2.30)	1.06E-02	2.03E-02	5.26 (2.85-9.71)	1.12E-07	2.59E-06	2.64 (1.67-4.18)	3.13E-05	2.66E-04
Gal-2	0.60 (0.42-0.85)	3.83E-03	1.04E-02	0.51 (0.33-0.79)	2.34E-03	3.98E-03	0.85 (0.57-1.27)	4.27E-01	5.73E-01
$G0^n$	1.13 (0.80-1.59)	4.89E-01	5.67E-01	0.69 (0.46-1.04)	7.34E-02	8.91E-02	0.60 (0.40-0.91)	1.65E-02	6.40E-02
$G1^n$	1.58 (1.07-2.34)	2.14E-02	3.42E-02	5.65 (3.02-10.58)	6.43E-08	2.59E-06	3.10 (1.87-5.14)	1.25E-05	1.56E-04
$G2^n$	0.68 (0.48-0.94)	2.12E-02	3.42E-02	0.58 (0.38-0.91)	1.69E-02	2.52E-02	1.01 (0.68-1.49)	9.63E-01	9.63E-01
nGal	0.88 (0.63-1.24)	4.67E-01	5.53E-01	1.44 (0.95-2.18)	8.37E-02	9.93E-02	1.64 (1.09-2.47)	1.76E-02	6.40E-02
F^{ntotal}	0.16 (0.08-0.35)	2.46E-06	2.51E-05	0.28 (0.14-0.53)	1.13E-04	2.88E-04	0.81 (0.51-1.27)	3.51E-01	5.03E-01
$FG0^{ntotal}/G0^n$	0.53 (0.32-0.86)	1.07E-02	2.03E-02	0.61 (0.39-0.94)	2.49E-02	3.52E-02	0.94 (0.65-1.37)	7.41E-01	8.05E-01
$FG1^{ntotal}/G1^n$	0.16 (0.07-0.34)	4.11E-06	3.33E-05	0.22 (0.10-0.47)	1.10E-04	2.88E-04	1.11 (0.76-1.62)	5.80E-01	6.72E-01
$FG2^{ntotal}/G2^n$	0.03 (0.01-0.14)	5.22E-06	3.33E-05	0.01 (0.00-0.08)	2.61E-06	1.33E-05	0.95 (0.66-1.38)	7.97E-01	8.30E-01
F^n	0.41 (0.26-0.64)	8.28E-05	3.84E-04	0.33 (0.19-0.57)	6.03E-05	1.71E-04	1.15 (0.79-1.68)	4.73E-01	6.03E-01
$FG0^n/G0^n$	0.77 (0.54-1.08)	1.32E-01	1.72E-01	0.67 (0.46-0.99)	4.25E-02	5.56E-02	1.03 (0.70-1.52)	8.84E-01	9.02E-01

$FG1^n/G1^n$	0.17 (0.08-0.38)	1.44E-05	8.16E-05	0.12 (0.05-0.28)	2.41E-06	1.33E-05	1.48 (1.01-2.15)	4.22E-02	1.19E-01
$FG2^n/G2^n$	0.03 (0.01-0.19)	1.04E-04	4.42E-04	0.01 (0.00-0.06)	1.95E-07	2.59E-06	1.12 (0.79-1.59)	5.28E-01	6.42E-01
FB^n	1.72 (1.22-2.43)	1.99E-03	5.97E-03	2.30 (1.40-3.80)	1.11E-03	2.02E-03	0.79 (0.55-1.14)	2.14E-01	3.77E-01
$FBG0^n/G0^n$	1.18 (0.85-1.64)	3.26E-01	3.95E-01	1.36 (0.93-1.98)	1.17E-01	1.35E-01	0.95 (0.64-1.39)	7.79E-01	8.28E-01
$FBG1^n/G1^n$	3.72 (1.84-7.51)	2.52E-04	9.89E-04	7.44 (3.05-18.12)	1.00E-05	3.64E-05	0.69 (0.48-0.99)	4.59E-02	1.23E-01
$FBG2^n/G2^n$	4.27 (1.55-11.78)	4.96E-03	1.19E-02	OR>1	2.77E-07	2.59E-06	0.68 (0.44-1.06)	9.09E-02	2.21E-01
FB^n/F^n	1.86 (1.28-2.73)	1.32E-03	4.49E-03	2.57 (1.50-4.42)	6.40E-04	1.21E-03	0.76 (0.53-1.09)	1.36E-01	2.79E-01
$FB^n/F^{n\ total}$	1.79 (1.25-2.55)	1.41E-03	4.49E-03	2.43 (1.46-4.04)	6.30E-04	1.21E-03	0.80 (0.55-1.16)	2.42E-01	4.11E-01
$F^n/(B^n + FB^n)$	0.73 (0.51-1.04)	8.44E-02	1.13E-01	0.31 (0.16-0.60)	4.87E-04	9.93E-04	0.88 (0.61-1.28)	5.13E-01	6.38E-01
$B^n/(F^n + FB^n)$	5.87 (2.49-13.87)	5.41E-05	2.76E-04	7.38 (2.50-21.75)	2.92E-04	6.47E-04	1.16 (0.79-1.71)	4.53E-01	5.92E-01
$FBG2^n/FG2^n$	OR>1	1.01E-02	2.03E-02	OR>1	9.81E-07	6.25E-06	0.92 (0.67-1.27)	6.30E-01	7.14E-01
$FBG2^n/(FG2^n + FBG2^n)$	6.89 (1.74-27.31)	5.99E-03	1.33E-02	OR>1	3.56E-07	2.59E-06	0.80 (0.57-1.10)	1.71E-01	3.12E-01
$FG2^n/(BG2^n + FBG2^n)$	0.19 (0.09-0.37)	1.16E-06	2.42E-05	0.02 (0.00-0.09)	2.91E-06	1.35E-05	0.43 (0.14-1.31)	1.37E-01	2.79E-01
$BG2^n/(FG2^n + FBG2^n)$	OR>1	5.07E-06	3.33E-05	OR>1	2.58E-05	8.38E-05	1.09 (0.74-1.59)	6.70E-01	7.43E-01
nFuc	0.27 (0.16-0.45)	4.69E-07	2.39E-05	0.15 (0.06-0.36)	2.63E-05	8.38E-05	0.76 (0.46-1.28)	3.05E-01	4.58E-01
nBis	2.05 (1.39-3.02)	3.13E-04	1.14E-03	2.41 (1.51-3.82)	2.01E-04	4.78E-04	0.80 (0.54-1.19)	2.71E-01	4.45E-01

NC: normal cognitive functioning; MCI: mild cognitive impairment.

[#] adjusting for the effects of age, sex, BMI, levels of education, history of malignant tumor, habit of salt intake, ischemic stroke, diabetes, hypertension and dyslipidemia (adjusting for the above effects other than age and sex for dementia *vs.* NC).

$p < 0.05$ was considered statistically significant.

**q* <0.05: significant after correction for FDR (False discovery rate).

Supplementary Table 6 The associations between normalized initia IgG *N*-glycans and AD

Glycans	Median (P_{25}-P_{75})		AOR (95% CI)	*p-adjusted*	*q**
	AD (*n*=47)	NC (*n*=81)			
GP1	0.35 (0.23-0.66)	0.05 (0.02-0.20)	7.31 (2.92-18.28)	2.16E-05	7.92E-05
GP2	0.58 (0.34-0.86)	0.28 (0.10-0.45)	1.94 (1.12-3.35)	1.74E-02	2.73E-02
GP4	39.43 (34.07-44.12)	42.02 (34.75-47.92)	0.85 (0.54-1.35)	4.97E-01	5.79E-01
GP5	0.22 (0.12-0.42)	0.00 (0.00-0.02)	37.98 (7.48-192.88)	1.15E-05	7.44E-05
GP6	6.57 (5.23-7.64)	6.22 (4.76-7.78)	1.17 (0.74-1.87)	5.00E-01	5.79E-01
GP7	0.22 (0.08-0.55)	0.01 (0.00-0.05)	6.54 (2.80-15.26)	1.43E-05	7.44E-05
GP8	14.63 (13.37-15.64)	11.53 (10.35-12.87)	5.27 (2.47-11.22)	1.69E-05	7.44E-05
GP9	4.25 (3.25-5.56)	2.97 (1.72-4.64)	2.02 (1.23-3.33)	5.77E-03	1.06E-02
GP10	2.26 (1.20-3.04)	0.73 (0.52-1.13)	5.78 (2.68-12.45)	7.50E-06	7.44E-05
GP11	0.10 (0.05-0.29)	0.08 (0.06-0.23)	1.39 (0.59-3.28)	4.54E-01	5.79E-01
GP12	0.36 (0.21-0.76)	0.14 (0.04-0.32)	2.56 (1.49-4.39)	6.31E-04	1.54E-03
GP13	0.44 (0.26-0.89)	0.14 (0.04-0.32)	5.79 (2.04-16.40)	9.46E-04	2.08E-03
GP14	10.68 (7.42-13.00)	13.73 (11.43-17.54)	0.39 (0.21-0.71)	2.31E-03	4.62E-03
GP15	0.50 (0.34-0.72)	0.11 (0.06-0.18)	9.36 (3.46-25.32)	1.08E-05	7.44E-05
GP16	2.85 (2.34-3.29)	3.97 (2.96-4.64)	0.33 (0.19-0.59)	1.63E-04	5.12E-04
GP17	0.80 (0.67-1.16)	0.83 (0.51-1.20)	1.09 (0.67-1.77)	7.29E-01	7.29E-01
GP18	6.95 (5.79-8.78)	10.52 (7.73-13.29)	0.28 (0.14-0.55)	1.90E-04	5.23E-04
GP19	1.76 (1.20-2.23)	1.76 (1.28-2.24)	0.73 (0.40-1.33)	3.04E-01	4.18E-01
GP21	0.31 (0.26-0.48)	0.23 (0.17-0.31)	1.59 (0.96-2.64)	7.42E-02	1.09E-01
GP22	0.15 (0.08-0.26)	0.07 (0.46-0.14)	3.73 (1.40-9.95)	8.47E-03	1.43E-02
GP23	0.79 (0.52-1.13)	0.67 (0.42-1.03)	0.90 (0.63-1.27)	5.42E-01	5.96E-01
GP24	0.73 (0.46-1.02)	0.82 (0.59-1.33)	1.08 (0.73-1.60)	7.05E-01	7.29E-01

*q <0.05: significant after correction for FDR (False discovery rate).
AD: Alzheimer’s disease; NC: normal cognitive functioning.
adjusting for the effects of age, sex, BMI, levels of education, history of malignant tumor, habit of salt intake, ischemic stroke, diabetes, hypertension and dyslipidemia.
$p < 0.05$ was considered statistically significant.

Supplementary Table 7 Regression coefficient of IgG *N*-glycans in dementia compared to NC group by Ridge and Stepwise based on logistic regression as well as Lasso regression

GPs	Ridge	Stepwise (Forward)	Stepwise (Backward)	Lasso
GP1	—	—	—	0.354
GP2	—	—	—	—
GP5	0.384	—	—	0.349
GP7	—	—	—	—
**GP8**	**0.823**	**1.757**	**1.559**	**0.883**
**GP9**	**0.346**	**0.721**	**0.589**	**0.204**
GP10	0.428	—	0.900	0.333
GP12	—	—	—	—
GP13	0.338	—	1.667	0.109
**GP14**	**-0.527**	**-1.534**	**-1.663**	**-0.453**
GP15	0.487	—	—	0.471
GP16	—	—	—	-0.019
GP18	-0.299	—	—	-0.283
GP22	—	—	—	—

NC: normal cognitive functioning; Numbers with the bold font stand for the intersection glycans selected through the above three selection methods. Ridge and Stepwise screening were based on Logistic regression.

Supplementary Table 8 Diagnostic performance (GP8+GP9+GP14) measured by using the 5-fold cross-validation random forest classifier

Groups	AUC (95% CI)	Sensitivity	Specificity	NMSE	MSE	MAE
GP8+GP9+GP14						
Dementia *vs.* NC	0.876 (0.815-0.923)	79.01	85.19	1.131	0.124	0.264
AD *vs.* NC	0.887 (0.819-0.936)	87.23	85.19	0.976	0.075	0.218
Dementia *vs.* MCI	0.815 (0.752-0.868)	69.14	85.19	3.373	0.224	0.401
MCI *vs.* NC	0.640 (0.568-0.709)	75.00	53.09	7.322	0.234	0.445

NC: normal cognitive functioning; MCI: mild cognitive impairment; AD: Alzheimer's disease; GP: Glycan Peak; AUC: Area Under Curve; CI: Confidence Interval; NMSE: Normalized Mean Square Error; MSE: Mean Squared Error; MAE: Mean Absolute Error.

Supplementary Table 9 Regression coefficient of IgG *N*-glycans in AD compared to NC group by Ridge and Stepwise based on logistic regression as well as Lasso regression

GPs	Ridge	Stepwise (Forward)	Stepwise (Backward)	Lasso
GP1	—	—	—	0.314
GP2	—	1.192	0.972	—
GP5	0.391	—	—	0.104
GP7	—	—	—	—
**GP8**	**0.873**	**2.259**	**2.443**	**0.349**
GP9	—	—	0.866	—
GP10	0.419	—	—	0.233
GP12	—	—	—	—
GP13	—	—	—	—
GP14	-0.601	—	-2.438	—
GP15	0.643	—	1.375	0.403
GP16	—	—	—	—
GP18	-0.413	—	—	-0.233
GP22	—	—	—	—

AD: Alzheimer's disease; NC: normal cognitive functioning; Numbers with the bold font stand for the intersection glycans selected through the above three selection methods. Ridge and Stepwise screening were based on Logistic regression.

Supplementary Table 10 Regression coefficient of IgG *N*-glycans in MCI compared to NC group by Ridge and Stepwise based on logistic regression as well as Lasso regression

GPs	Ridge	Stepwise (Forward)	Stepwise (Backward)	Lasso
**GP1**	**0.594**	**1.451**	**1.547**	**0.390**
GP2	—	—	—	—
GP5	0.526	—	—	0.224
GP7	—	—	—	0.279
**GP10**	**0.504**	**0.811**	**0.992**	**0.068**
GP11	—	—	—	—
GP13	—	—	—	—
GP14	—	—	—	—
GP15	0.305	—	—	—
GP18	—	—	—	—
GP22	—	—	1.151	—
GP23	—	—	—	—

MCI: mild cognitive impairment; NC: normal cognitive functioning; Numbers with the bold font stand for the intersection glycans selected through the above three selection methods. Ridge and Stepwise screening were based on Logistic regression.

Supplementary Table 11 Regression coefficient of IgG *N*-glycans in Dementia compared to MCI group by Ridge and Stepwise based on logistic regression as well as Lasso regression

GPs	Ridge	Stepwise (Forward)	Stepwise (Backward)	Lasso
**GP8**	**0.013**	**0.646**	**0.781**	**0.361**
GP9	0.008	—	—	0.135
GP11	-0.006	—	—	—
**GP12**	**0.010**	**0.773**	**0.774**	**0.272**
GP16	-0.015	—	—	-0.260
**GP18**	**-0.015**	**-1.557**	**-1.813**	**-0.758**
GP21	0.009	—	—	0.155
**GP23**	**0.012**	**0.949**	**1.082**	**0.522**

MCI: mild cognitive impairment; Numbers with the bold font stand for the intersection glycans selected through the above three selection methods. Ridge and Stepwise screening were based on Logistic regression.

Supplementary Table 12 Regression coefficient of IgG *N*-glycans in AD compared to MCI group by Ridge and Stepwise based on logistic regression as well as Lasso regression

GPs	Ridge	Stepwise (Forward)	Stepwise (Backward)	Lasso
**GP8**	**0.176**	**0.764**	**0.810**	**0.509**
GP9	—	—	—	—
GP10	0.160	—	—	0.228
GP11	—	—	—	—
**GP12**	**0.192**	**1.166**	**1.393**	**0.677**
GP16	-0.227	—	—	-0.277
**GP18**	**-0.258**	**-2.491**	**-2.670**	**-1.512**
GP21	0.129	—	—	0.253
**GP23**	**0.198**	**1.616**	**1.882**	**1.039**

AD: Alzheimer’s disease; MCI: mild cognitive impairment; Numbers with the bold font stand for the intersection glycans selected through the above three selection methods. Ridge and Stepwise screening were based on Logistic regression.

Supplementary Table 13 Diagnostic performance illustrated by using the 5-fold cross-validation random forest classifier for every two groups

Groups	AUC (95% CI)	Sensitivity	Specificity	NMSE	MSE	MAE
Dementia *vs.* NC (GP8+GP9+GP14)	0.876 (0.815-0.923)	79.01	85.19	1.131	0.124	0.264
AD *vs.* NC (GP8)	0.703 (0.616-0.781)	89.36	56.79	1.752	0.249	0.341
Dementia *vs.* MCI (GP8+GP12+GP18+GP23)	0.842 (0.782-0.891)	88.89	67.59	2.608	0.167	0.348
MCI *vs.* NC (GP1+GP10)	0.809 (0.746-0.863)	74.07	80.25	1.613	0.178	0.306
AD *vs.* MCI (GP8+GP12+GP18+GP23)	0.917 (0.862-0.955)	95.74	82.41	2.116	0.125	0.263

NC: normal cognitive functioning; MCI: mild cognitive impairment; AD: Alzheimer's disease; GP: Glycan Peak; AUC: Area Under Curve; CI: Confidence Interval; NMSE: Normalized Mean Square Error; MSE: Mean Squared Error; MAE: Mean Absolute Error.
